# Cardiac pericytes mediate the remodeling response to myocardial infarction

**DOI:** 10.1172/JCI162188

**Published:** 2023-05-15

**Authors:** Pearl Quijada, Shuin Park, Peng Zhao, Kamal S.S. Kolluri, David Wong, Kevin D. Shih, Kai Fang, Arash Pezhouman, Lingjun Wang, Ali Daraei, Matthew D. Tran, Elle M. Rathbun, Kimberly N. Burgos Villar, Maria L. Garcia-Hernandez, Thanh T.D. Pham, Charles J. Lowenstein, M. Luisa Iruela-Arispe, S. Thomas Carmichael, Eric M. Small, Reza Ardehali

**Affiliations:** 1 Department of Integrative Biology and Physiology,; 2 Eli and Edythe Broad Stem Research Center,; 3 Molecular Biology Institute,; 4 Molecular, Cellular, and Integrative Physiology Graduate Program, and; 5 Cardiology, Internal Medicine, David Geffen School of Medicine, UCLA, Los Angeles, California, USA.; 6 Section of Cardiology, Department of Internal Medicine, Baylor College of Medicine, Houston, Texas, USA.; 7 The Texas Heart Institute, Houston, Texas, USA.; 8 Cardiology, Second Affiliated Hospital, School of Medicine, Zhejiang University, Hangzhou, China.; 9 Department of Neurology, David Geffen School of Medicine, UCLA, Los Angeles, California, USA.; 10 Department of Pathology,; 11 Department of Medicine, Aab Cardiovascular Research Institute, School of Medicine and Dentistry, and; 12 Allergy/Immunology and Rheumatology, University of Rochester, Rochester, New York, USA.; 13 Division of Cardiology, Department of Medicine, Johns Hopkins University School of Medicine, Maryland, USA.; 14 Department of Cell and Developmental Biology, Feinberg School of Medicine, Northwestern University, Chicago, Illinois, USA.; 15 Department of Pharmacology and Physiology and; 16 Department of Biomedical Engineering, University of Rochester, Rochester, New York, USA.

**Keywords:** Cardiology, Vascular Biology, Cardiovascular disease, Fibrosis, Pericytes

## Abstract

Despite the prevalence of pericytes in the microvasculature of the heart, their role during ischemia-induced remodeling remains unclear. We used multiple lineage-tracing mouse models and found that pericytes migrated to the injury site and expressed profibrotic genes, coinciding with increased vessel leakage after myocardial infarction (MI). Single-cell RNA-Seq of cardiac pericytes at various time points after MI revealed the temporally regulated induction of genes related to vascular permeability, extracellular matrix production, basement membrane degradation, and TGF-β signaling. Deleting TGF-β receptor 1 in chondroitin sulfate proteoglycan 4–expressing (*Cspg4*-expressing) cells reduced fibrosis following MI, leading to a transient improvement in the cardiac ejection fraction. Furthermore, genetic ablation of *Cspg4*-expressing cells resulted in excessive vascular permeability, a decline in cardiac function, and increased mortality in the second week after MI. These data reveal an essential role for cardiac pericytes in the control of vascular homeostasis and the fibrotic response after acute ischemic injury, information that will help guide the development of novel strategies to preserve vascular integrity and attenuate pathological cardiac remodeling.

## Graphical abstract

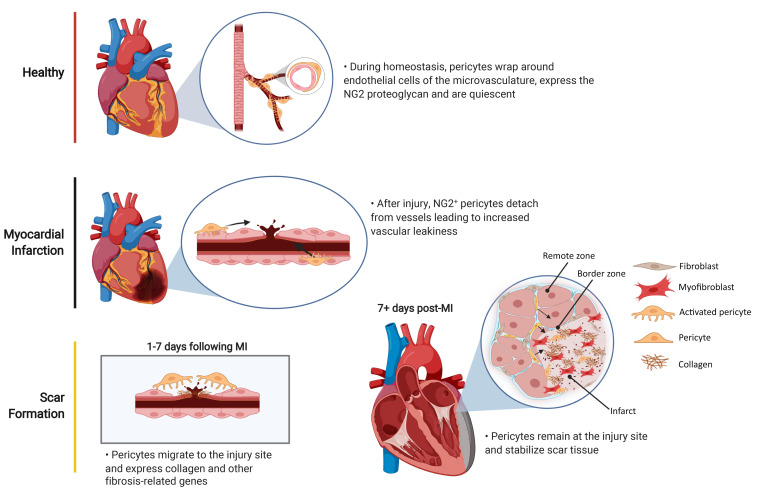

## Introduction

Injury to the adult mammalian heart results in irreversible loss of cardiomyocytes and triggers an inflammatory cascade that forms a collagen-rich scar. A significant component of fibrosis following myocardial infarction (MI) is the conversion of fibroblasts to myofibroblasts (1), which synthesize and secrete excessive extracellular matrix (ECM) proteins (2). However, the heart lacks the regenerative capacity to replace scar tissue with healthy myocardium, leading to a decline in cardiac function and heart failure. Although targeting cardiac myofibroblast conversion may be a therapeutic goal for treating heart disease, it is unclear whether vascular mural cells, such as pericytes, play a role in developing cardiac fibrosis.

Pericytes are an understudied population of vascular mural cells but have recently garnered attention because of the reported unexpected contribution to fibrotic disease progression in several organs (3–5). Under homeostatic conditions, pericytes are tightly associated with endothelial cells (ECs) of the microvasculature (6), where they maintain vascular integrity and vessel contractility (7). In contrast, a deficit of pericyte coverage over the vasculature results in capillary loss and vascular leakage in the retina, brain, and kidney (8–10). Several studies have shown that pathological stimuli such as ischemia promote pericyte dissociation from capillaries, induction of ECM-related gene programs, and differentiation toward a myofibroblast-like fate following spinal cord injury or kidney ischemia (11, 12). In contrast, reducing pericyte proliferation results in less fibrosis after spinal cord damage (13). Although isolated pericytes exhibit mesenchymal cell characteristics during in vitro culture (14), the adoption of a conclusive mesenchymal cell fate by pericytes in vivo has been challenged (15). Studies have demonstrated that Gli1^+^ perivascular mesenchymal cells contribute to cardiac fibrosis by directly converting into myofibroblasts, and their depletion improves cardiac function during pressure overload due to reduced fibrosis (16), suggesting that nonfibroblasts support the development of cardiac fibrosis. Additionally, epithelial-mesenchymal conversion during cardiac development generates mostly resident cardiac fibroblasts (17, 18). Their transition to a myofibroblast phenotype is primarily mediated by TGF-β signaling and Wnt/β-catenin signaling (19–21). However, investigating these integral pathways in perivascular cells, such as cardiac pericytes, has not to our knowledge been previously explored. Therefore, as the profibrotic role of pericytes is observed across multiple organs, there is a need to determine whether cardiac pericytes also contribute to fibrosis during MI.

In this study, we hypothesized that cardiac pericytes participate in vascular and fibrotic remodeling during ischemic damage in the heart. Here, we used the chondroitin sulfate proteoglycan 4 (*Cspg4*) gene marker to reliably label and lineage trace pericytes in the adult heart following MI (22). Our results demonstrated that, following pathological injury to the heart, pericytes detached from the vasculature, proliferated, and migrated to the injury site, producing ECM and contributing to cardiac fibrosis. Single-cell RNA-Seq (scRNA-Seq) of pericytes at different post-MI time points revealed transcriptional changes consistent with increased migration and loss of cell-to-cell adhesion markers during the early phases of MI, followed by a significant increase in gene programs related to vascular permeability, ECM organization, and fibrosis, specifically genes related to the TGF-β signaling pathway. Disruption of TGF-β signaling in pericytes by deletion of *Tgfbr1* in the *Cspg4*-expressing cell lineage resulted in a modest and transient improvement in cardiac function 1 week after MI, as well as a reduction in myocardial fibrosis 2 weeks after MI. Finally, genetic ablation of pericytes induced vascular leakage in the myocardium following MI, resulting in a rapid decline in cardiac ejection fraction and mouse survival. Our data show that cardiac vascular mural cells contributed to the post-MI fibrotic and vascular remodeling response.

## Results

### Defining Cspg4-lineage cardiac pericytes.

**Figure 1:**
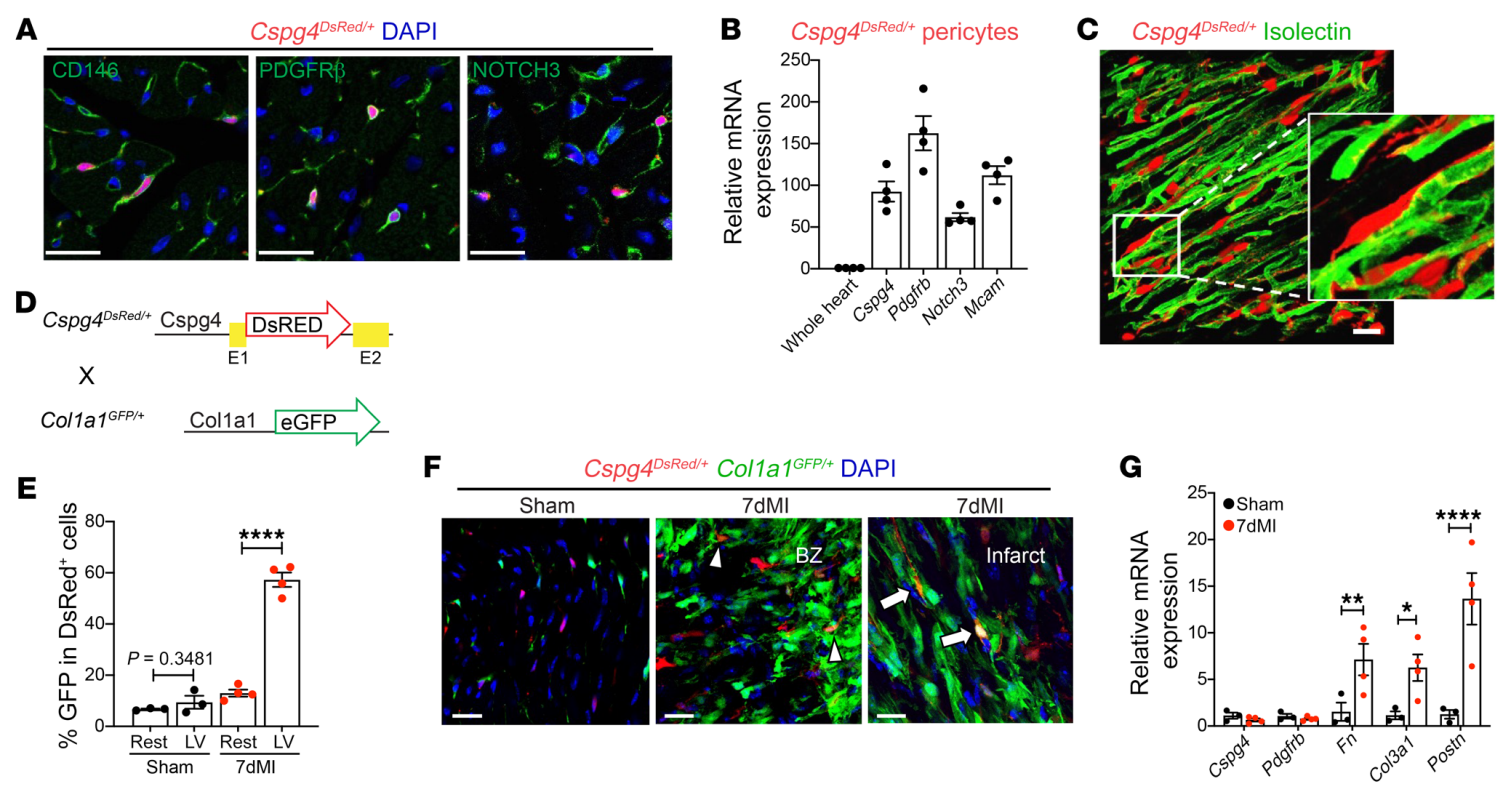
*Cspg4*^+^ cardiac pericytes express *Col1a1* after MI. (**A**) DsRed^+^ cells (red) in *Cspg4^DsRed/+^* hearts expressed the pericyte markers CD146, PDGFRβ, and Notch3 (markers are shown in green). Images are representative of 3 experiments. (**B**) Pericyte marker genes were highly expressed in DsRed^+^ cells isolated by FACS compared with expression in whole, unsorted heart samples. *n* = 4 hearts. (**C**) DsRed^+^ pericytes were closely associated with isolectin^+^ vasculature in healthy *Cspg4^DsRed/+^* hearts. Images are representative of 3 experiments. (**D**) Experimental strategy for the generation of *Cspg4^DsRed/+^* *Col1a1^GFP/+^* double-transgenic mice. (**E**) The percentage of DsRed^+^ pericytes that coexpressed GFP in *Cspg4^DsRed/+^* *Col1a1^GFP/+^* hearts increased in the left ventricle (LV) seven days after MI compared with sham-operated control hearts. *n* =3 sham and *n* = 4 MI hearts. An unpaired, 2-tailed Student’s *t* test was conducted to compare sham and 7-day post-MI (7dMI) hearts (rest and LV). (**F**) DsRed^+^GFP^+^ cells were observed in the BZ and infarct areas in *Cspg4^DsRed/+^* *Col1a1^GFP/+^* hearts. Arrowheads indicate DsRed^+^ cells with low GFP expression, and arrows represent DsRed^+^ cells with high expression of GFP. Images are representative of 3 sham and 4 MI experiments. (**G**) Relative gene expression in DsRed^+^ cells collected by FACS and analyzed following RT-qPCR. *n* = 3 sham and *n* = 4 MI hearts. An unpaired, 2-tailed Student’s *t* test was conducted to compare sham and 7dMI samples for each gene. Scale bars: 20 μm. *P < 0.05, **P < 0.01, and ****P < 0.0001.

Expression of the *Cspg4* gene has been reported in vascular mural cells (22, 23) and microvasculature-associated cells in the adult myocardium (24). To explore the specificity of this gene for identification of pericytes in the adult heart, we confirmed the endogenous colocalized expression of the protein neuron-glial antigen 2 (NG2, encoded by the *Cspg4* gene) with canonical proteins expressed in pericytes such as CD146 and PDGFRβ using immunofluorescence (IF) staining and flow cytometric analyses (Supplemental Figure 1, A and B; supplemental material available online with this article; https://doi.org/10.1172/JCI162188DS1). As NG2 proved an accurate marker of cardiac pericytes, we used the *Cspg4*-DsRed.T1–transgenic mouse strain (referred to hereafter as *Cspg4^DsRed/+^*), in which the DsRed.T1 expression cassette is under the control of the *Cspg4* promoter (25). The *Cspg4^DsRed/+^* mouse model did not label cardiomyocytes, fibroblasts, leukocytes, or ECs (Supplemental Figure 1, C–E) but instead identified cells that coexpressed pericyte markers (CD146, PDGFRβ, and NOTCH3), as confirmed by IF staining and real-time quantitative PCR (RT-qPCR) of isolated *Cspg4-DsRed^+^* cells using FACS (Figure 1, A and B). Consistent with previous reports, we found that *Cspg4-DsRed^+^* cells were closely associated with cardiac microvasculature in healthy myocardium (24) (Figure 1C). We also found *Cspg4-DsRed^+^* cells that coexpressed α smooth muscle actin (αSMA) around larger arteries, with approximately 20% overlap in the aorta and arteries (Supplemental Figure 1F). However, this expression pattern was distinct from that of pericytes diffusely present in the cardiac interstitial area (Supplemental Figure 1F).

### Cspg4^+^ pericytes express ECM proteins at the site of ischemic injury.

More recently, we showed that mural cells exhibit phenotypic changes in response to brain and heart ischemia (5). To determine whether *Cspg4*^+^ cells express ECM components, such as collagens, we generated a reporter mouse strain by crossing *Cspg4^DsRed/+^* mice with *Col1a1*^GFP/+^ mice, in which the collagen1a1 (Col1a1) promoter drives the expression of GFP (hereafter referred to as *Cspg4^DsRed/+^* *Col1a1^GFP/+^*) (Figure 1D). In the whole heart, we detected GFP expression in DsRed^+^ cells around cardiac valves, whereas following MI, GFP expression was noticeable in DsRed^+^ cells found in the border zone (BZ) and infarct regions (Supplemental Figure 1, G and H). We observed a significant increase in cells that coexpressed *Col1a1-GFP^+^* and *Cspg4-DsRed* seven days after MI (Supplemental Figure 1I), particularly in the left ventricular infarct areas (Figure 1, E and F, and Supplemental Figure 1J). We further verified that FACS-isolated *Cspg4-DsRed^+^* cells had elevated expression of *Fn*, *Col13a1*, and *Postn* compared with expression levels in sham-isolated pericytes (Figure 1G). However, we did not observe reduced expression of *Cspg4* or *Pdgfrb* in pericytes collected from *Cspg4*-*DsRed* mice Figure 1G), suggesting that our pericyte reporter mouse had cells that maintained their pericyte identity.

### Cspg4-lineage–traced pericytes express profibrotic genes following MI.

**Figure 2:**
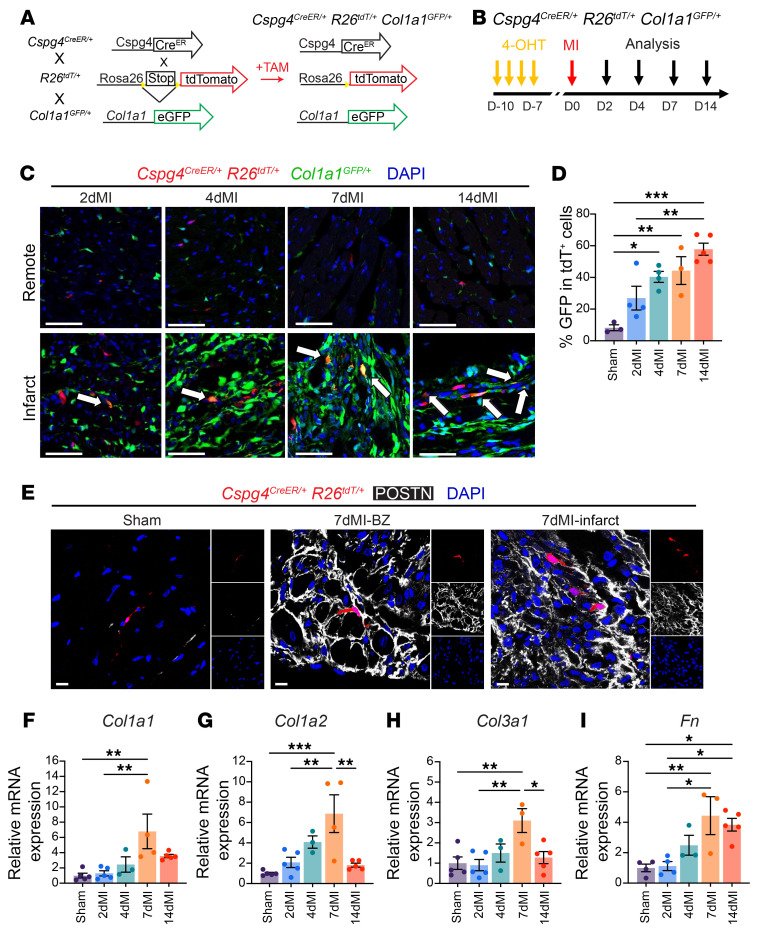
*Cspg4*-lineage pericytes accumulate in the infarct area and express *Col1a1* after MI. (**A**) Breeding plan for the generation of *Cspg4^CreER/+^* *Rosa26^tdT/+^* *Col1a1^GFP/+^* triple-transgenic mice. (**B**) Schematic for TAM injection before MI. Hearts were analyzed 2, 4, 7, and 14 days (D) after MI. (**C**) Representative images of the left ventricular free wall of *Cspg4^CreER/+^* *Rosa26^tdT/+^* *Col1a1^GFP/+^* hearts during MI. tdTomato^+^GFP^+^ cells were observed in the infarct region (bottom) compared with the remote region (top). Scale bars: 50 μm for both infarct and remote regions. Images are representative of 4 2dMI, 4 4dMI, 3 7dMI, and 5 14dMI experiments. Arrows highlight tdTomato^+^GFP^+^ cells. (**D**) GFP expression in tdTomato^+^ (tdT^+^) cells increased over the MI period as analyzed by flow cytometry. *n* = 3 sham, *n* = 4 2dMI, *n* = 4 4dMI, *n* = 3 7dMI, and *n* = 5 14dMI hearts. (**E**) POSTN was expressed in tdTomato^+^ lineage–traced pericytes following 7 days of MI in the BZ and infarct areas but not in noninjured hearts of the sham-operated mice. Scale bars: 20 μm for infarct and remote regions. Images are representative of 3 sham and 3 7dMI experiments. (**F**–**I**) Relative gene expression of profibrotic and ECM genes in tdTomato^+^ cells collected via FACS from sham and MI hearts. *n* = 4–5 sham, *n* = 4–5 2dMI, *n* = 3 4dMI, *n* = 3–4 7dMI, and *n* = 5 14dMI hearts. *P < 0.05, **P < 0.01, and ***P < 0.001, by 1-way ANOVA with Tukey’s multiple-comparison test (**D** and **F**–**I**).

The constitutive labeling strategy of the *Cspg4^DsRed/+^* mouse model raises the possibility of marking other cardiac cells as a result of transient expression of *Cspg4* in response to injury. Alternatively, *Cspg4*-dependent DsRed expression may be reduced in pericytes that transform toward a fibrotic phenotype. To circumvent these limitations, we generated *Cspg4^CreER/+^* *Rosa26^tdT/+^* mice that allow for tamoxifen-dependent (TAM-dependent) temporal control over *Cspg4*-based labeling, such that pericytes expressing *Cspg4* can be marked at specific time points to track their location, fate, and gene expression profiles at single-cell resolution during homeostasis and following experimental injury. Pericyte labeling in the *Cspg4^CreER/+^* *Rosa26^tdT/+^* mouse strain was confirmed by colabeling tdTomato cells for pericyte markers (*Cspg4*, *Pdgfrb*, *Mcam*, *Notch3*) using IF staining, RT-qPCR, and flow cytometric analyses (Supplemental Figure 2, A–E). It is unknown whether *Cspg4*-lineage pericytes express collagen or if other cell types, such as collagen-producing fibroblasts, begin upregulating *Cspg4* expression after injury. Therefore, labeling cardiac pericytes before the injury and tracking their fate after MI is essential. To address this question, *Cspg4^CreER/+^* *Rosa26^tdT/+^* mice were further crossed with *Col1a1^GFP/+^* mice (hereafter referred to as *Cspg4^CreER/+^* *Rosa26^tdT/+^* *Col1a1^GFP/+^*) to evaluate the contribution of lineage-traced pericytes to the production of collagen (26) (Figure 2A). *Cspg4^CreER/+^* *Rosa26^tdT/+^* *Col1a1^GFP/+^* mice received TAM for 4 days, followed by either sham or MI surgery 1 week later, and hearts were harvested at post-MI days 2, 4, 7, and 14 (Figure 2B). Before analyzing the injured hearts, we confirmed that expression of *Col1a1-GFP* was not detectable in healthy cardiac pericytes localized within the left ventricles and was only found to be expressed in the lining of the aorta (Supplemental Figure 2F). Following MI, tdTomato^+^ pericytes had progressively increased their expression of *Col1a1-GFP* in the BZ and infarct regions, as shown by confocal microscopy and quantified by flow cytometry (Figure 2, C and D). Seven days after MI, we detected the expression of periostin (POSTN), a protein upregulated in myofibroblasts (27), in pericytes located throughout the BZ and infarct regions (Figure 2E). In contrast, pericytes in sham hearts did not express POSTN (Figure 2E). Additionally, tdTomato^+^ pericytes showed increased expression of *Col1a1*, *Col1a2*, *Col3a1*, and *Fn1* at post-MI day 7 compared with sham and 2-day post-MI pericytes (Figure 2, F–I). These data strongly support that ischemic injury stimulates pericytes to acquire a profibrotic phenotype, most significantly 7 days after MI.

### Cspg4-lineage pericytes do not transdifferentiate into other cardiac cell types after MI.

Recent reports show that cardiac pericytes do not transdifferentiate into different cell types after transaortic constriction (TAC), challenging the idea that pericytes serve as progenitors for mesenchymal cell types (15). However, the TAC model involves different pathophysiology and results in diffuse fibrosis, unlike the replacement fibrosis observed after MI (28). We first compared the expression of profibrotic and ECM genes in *Cspg4*-lineage^+^ compared with *Cspg4*-lineage^–^ CD31^–^ cell populations and found that pericytes had upregulated levels of *Postn*, *Fn*, *Col1a1*, and *Col3a1* following 15 days of MI, but not after 28 days of TAC (Supplemental Figure 3, A–D). Our data indicate that ischemic injury is a potent trigger of the profibrotic phenotype in pericytes, but not TAC as previously described (15). Cspg4-lineage pericytes isolated from sham-operated hearts also displayed enriched expression of the pericyte genes *Cspg4*, *Pdgfrb*, and *Tbx18* (Supplemental Figure 3, E–G). However, these gene markers decreased in pericytes isolated 7 days after MI, indicating that cells may lose some pericyte identity before transformation to a profibrotic cell state (Supplemental Figure 3, E–G). The decreased expression of pericyte genes in isolated cells from the *Cspg4* lineage compared with the *Cspg4-DsRed* mouse provides a strong rationale for an inducible labeling approach of cardiac pericytes. Next, we sought to determine whether pericytes exhibit progenitor cell–like properties and transdifferentiate in vivo following myocardial ischemia. We did not observe protein marker expression representing each significant cardiac cell type (cardiomyocytes, resident fibroblasts, immune cells, or ECs) in cardiac pericytes subjected to MI (Supplemental Figure 3H). These results indicate that pericytes do not exhibit multipotent progenitor cell characteristics following MI, consistent with left ventricular pressure overload studies.

### Cardiac pericytes dissociate from the microvasculature and migrate to the site of injury after MI.

**Figure 3:**
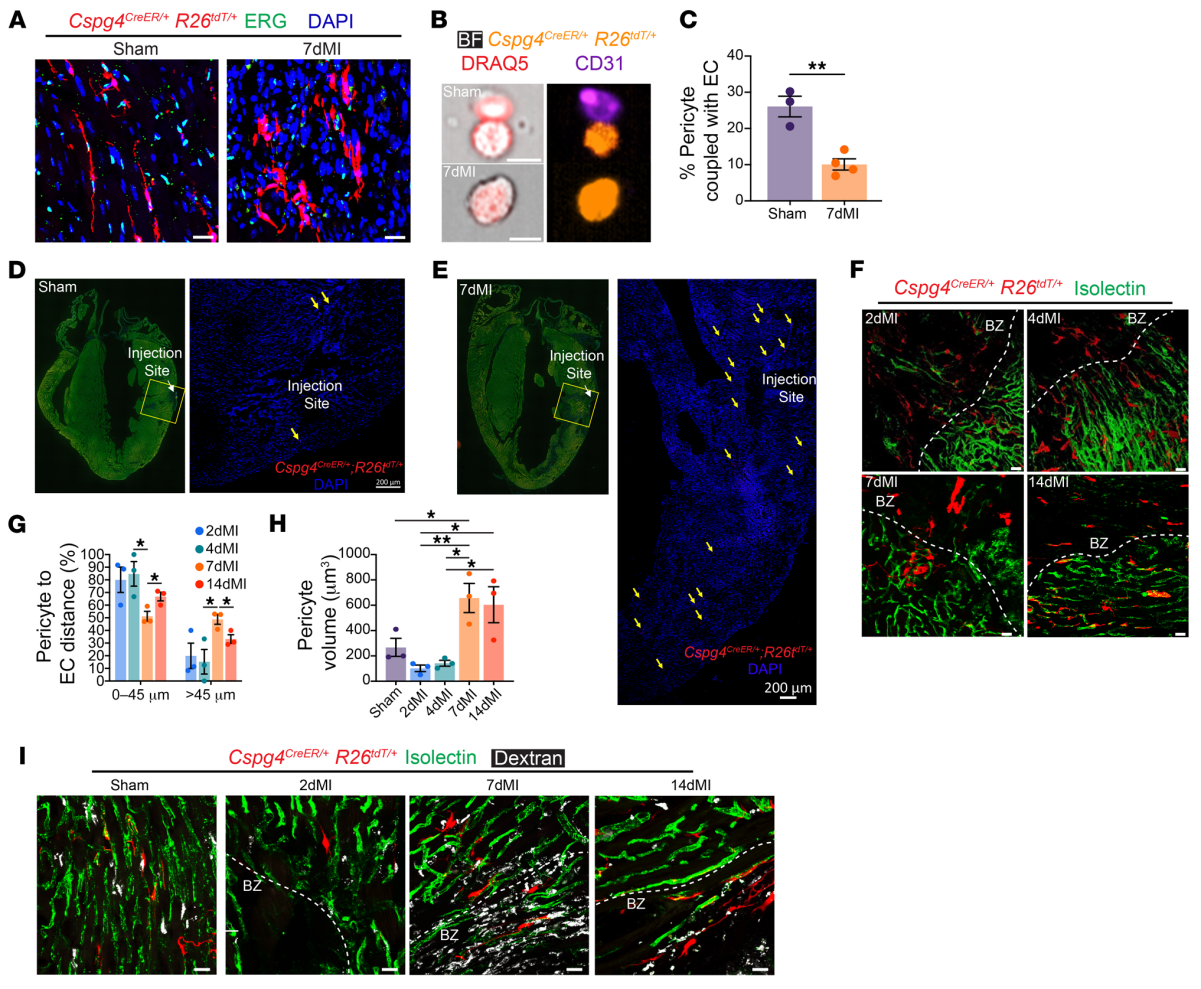
Cardiac pericytes dissociate from the microvasculature following MI. (**A**) Using IHC, the evaluation of *Cspg4*-lineage cells (red) was relative to ECs identified by the nuclear protein marker ERG (green) 7 days after infarction. Scale bars: 20 μm. (**B**) Analysis of pericyte-EC interactions using Amnis ImageStream Technology following sham surgery or 7 days of MI. Pericytes are labeled in orange, ECs are labeled with CD31 antibody (purple), and nucleated cells were visualized by staining with DRAQ5 (red) and overlayed with bright-field images. Scale bars: 10 μm. Images in **A** and **B** are representative of 3 sham and 4 7dMI experiments. (**C**) Pericyte-EC interactions were reduced in *Cspg4*-lineage mice following 7 days of MI. *n* = three sham and *n* = four 7dMI hearts. An unpaired, 2-tailed Student’s *t* test was performed to compare sham and 7dMI hearts. (**D**) Sham and (**E**) 7dMI *Cspg4^CreER/+^* *R26^tdT/+^* mice were subjected to an intramyocardial injection of 2 μL TAM immediately followed by surgery. (**D**) tdTomato^+^ pericytes were observed near the injection site in the sham-operated animals, whereas (**E**) tdTomato^+^ pericytes were discovered proximal and distal to the injection site following 7 days of MI. Yellow arrows indicate tdTomato^+^ cells. Scale bars: 200 μm. Images in **D** and **E** are representative of 3 sham and MI experiments each. (**F**) Visualization of pericytes relative to intact microvasculature in MI-injured hearts around the BZ regions. Isolectin (intact vasculature) is shown in green. Scale bars: 20 μm. (**G**) Percentage of pericytes in regions proximal (0–45 μm) or distal to the BZ area (>45 μm) following a time course of MI. *n* = 3 each for sham, 2dMI, 4dMI, 7dMI, and 14dMI hearts. Data were analyzed by 1-way ANOVA with Tukey’s multiple-comparison test. (**H**) Pericyte volume (μm^3^) was increased at post-MI days 7 and 14. *n* = 3 sham, 2dMI, 4dMI, 7dMI and 14dMI hearts. Data were analyzed by 1-way ANOVA with Tukey’s multiple-comparison test. (**I**) Visualization of pericytes relative to intact and leaky microvasculature in healthy (sham-treated) or MI-injured hearts around the BZ regions. Isolectin (intact vasculature, green) and dextran (leaky vasculature, white) was administered to mice on the day of isolation. Scale bars: 20 μm. Data in **F** and **I** are representative of 3 sham, 2dMI, 4dMI, 7dMI, and 14dMI experiments. *P < 0.05 and **P < 0.01.

One of the essential features of pericytes is their close association with the microvasculature during homeostasis (7). To investigate the interactions of *Cspg4*-lineage pericytes with ECs under acute ischemic stress, *Cspg4^CreER/+^ Rosa26^tdT/+^* and *Cspg4^CreER/+^* *Rosa26^mTmG/+^* (pericytes labeled with membrane-tethered GFP) mice underwent experimental MI surgery, and hearts were analyzed 7 days later (Supplemental Figure 4A). We used *Cspg4^CreER/+^* *Rosa26^mTmG/+^* mice as an independent transgenic mouse model to validate the findings observed in the *Cspg4^CreER/+^* *Rosa26^tdT/+^* mouse strain and exclude any inaccuracies that may have arisen from the use of a single lineage-tracing system. In both mouse models, pericytes dissociated from ETS-related gene^+^ (ERG^+^) ECs (ERG is an endothelial-specific transcription factor) (29) following ischemic injury as compared with sham controls imaged by confocal microscopy (Figure 3A and Supplemental Figure 4B). Consistent with our in vivo imaging analysis, *Cspg4*-lineage–traced pericytes showed decreased association with CD31^+^ ECs 7 days after MI as confirmed by flow cytometry (Supplemental Figure 4, C and D). These observations were further confirmed by visualization and quantification using Amnis ImageStream technology, which revealed decreased interaction between tdTomato^+^ pericytes and CD31^+^ ECs after MI (Figure 3, B and C).

Next, to assess whether pericytes undergo migration following dissociation from the microvasculature in response to ischemic injury, *Cspg4^CreER/+^* *Rosa26^tdT/+^* mice were administered a small amount of TAM in the left ventricular free wall followed by 7 days of sham or MI surgery (Supplemental Figure 4E). Sham hearts showed local labeling of pericytes at the site of the TAM injection (Figure 3D). Interestingly, in the MI hearts, we detected an abundance of labeled pericytes that migrated from the initial site of TAM injection toward the infarct and BZ regions (Figure 3E). This experiment demonstrates that cardiac pericytes can disperse from their original location after cardiac injury. To dissect the association of *Cspg4*-lineage–traced pericytes with the microvasculature, we next analyzed tdTomato^+^ cells relative to isolectin^+^ vasculature in the infarct and peri-infarct regions. Pericyte distance to the closest vascular bed in the uninjured hearts was virtually zero (i.e., pericytes were intimately embedded within the microvascular basement membrane) (Supplemental Figure 4F). In contrast, the distance between pericytes and the vasculature increased 7 days after MI compared with earlier time points (Figure 3, F and G). To gain insight into how quickly pericytes detach from blood vessels after an injury, we next performed a time course analysis of pericyte distribution after MI. We observed that pericytes responded to injury by locally detaching from the vasculature in the first few hours after MI (Supplemental Figure 4F). However, we saw the most significant increase in the number of pericytes at the site of injury following 7 days of MI, which was accompanied by substantial changes in pericyte morphology as evidenced by increases in cell volume, and cell area and a reduction in cell sphericity (Figure 3H and Supplemental Figure 4, G and H). Before harvesting the hearts, we infused mice with dextran to investigate whether pericyte detachment from the vasculature increases vascular permeability. Consistent with pericyte detachment from the vasculature, we observed increased vascular leakage in the infarcted regions of hearts after 7 days and 14 days of MI, but not at earlier MI time points (Figure 3I and Supplemental Videos 1 and 2). Our data suggest that cardiac pericytes play a prominent role in the injury response by dissociating from the microvasculature, migrating toward the infarct regions and contributing to the fibrotic response.

### Cspg4-lineage pericytes accumulate in the infarct region after MI by increased proliferation.

**Figure 4:**
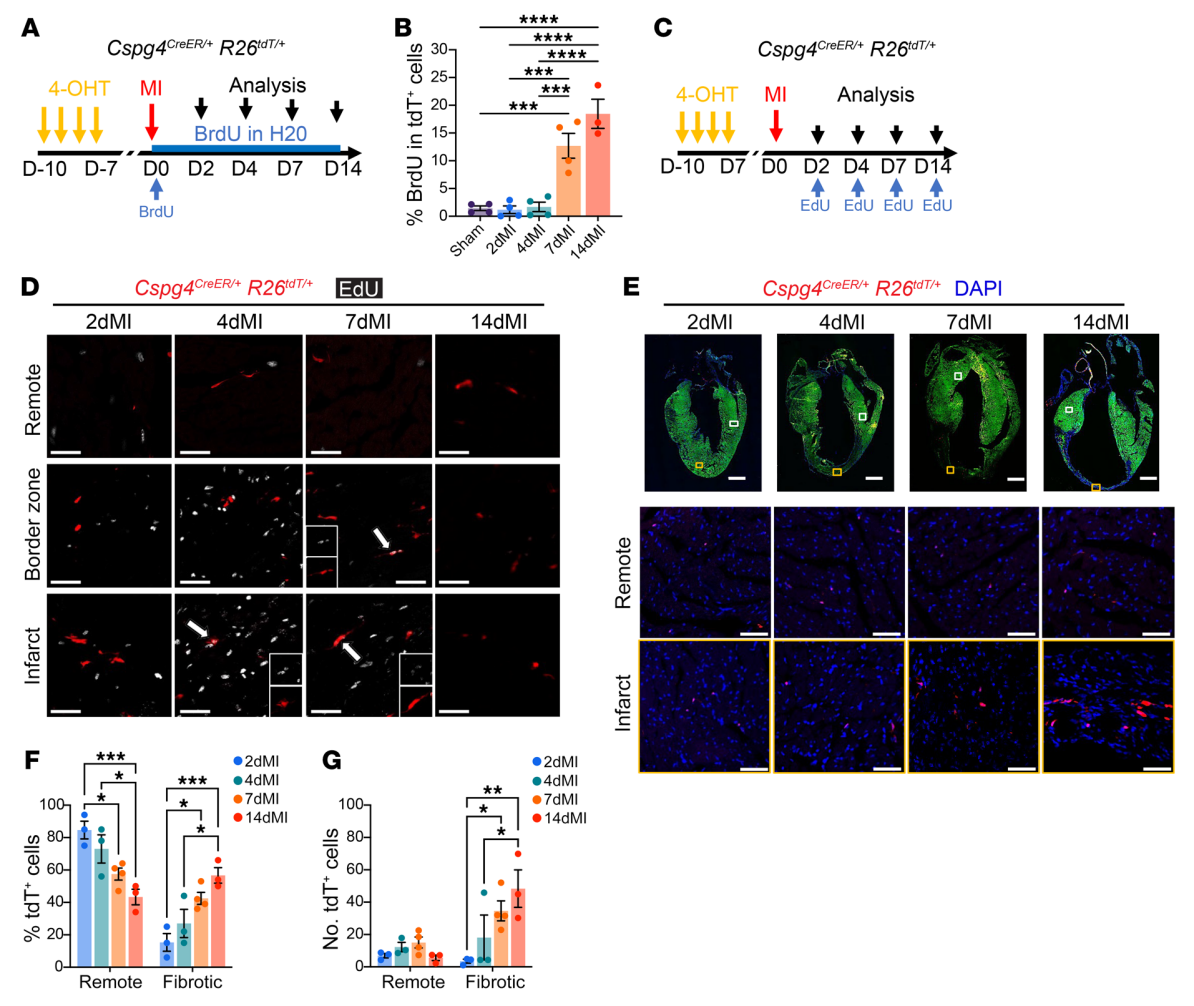
*Cspg4*-lineage cardiac pericytes proliferate in the infarcted area. (**A**) Schematic of BrdU administration. After surgery, *Cspg4^CreER/+^* *Rosa26^tdT/+^* mice received a single injection of BrdU, followed by ad libitum administration of water containing BrdU. (**B**) Flow cytometric analysis of the percentage of BrdU^+^tdTomato^+^ cells in *Cspg4^CreER/+^* *Rosa26^tdT/+^* mouse hearts that had undergone sham or MI operation. *n* = 4 sham, 2dMI, 4dMI, 7dMI hearts and *n* = 3 14dMI hearts. Data were analyzed by 1-way ANOVA with Tukey’s multiple-comparison test. (**C**) Schematic of *Cspg4^CreER/+^* *Rosa26^tdT/+^* mice subjected to single injections of EdU for 4 hours at specified MI time points. Analyses are representative of four 2dMI, 4dMI, and 7dMI experiments, and three 14dMI experiments. (**D**) Proliferating (EdU^+^, white) tdTomato^+^ cells were found within the BZ and infarct area (arrows) but not in the remote area. Scale bars: 50 μm. (**E**) Representative images show the localization of tdTomato^+^ cells in the remote and infarct areas during MI. Scale bars: 50 μm (remote and infarct regions) and 1 mm (whole heart images). Images are representative of four 2dMI, 4dMI, 14dMI experiments, and three 7dMI experiments. The (**F**) percentage and (**G**) number of tdTomato^+^ cells in the fibrotic regions of the heart increased during MI and relative to the remote region. *n* = 3 sham, 2dMI, 4dMI, and 14dMI hearts and *n* = four 7dMI hearts. Data were analyzed by 1-way ANOVA with Tukey’s multiple-comparison test. *P < 0.05, **P < 0.01, ***P < 0.001, and ****P < 0.0001.

To determine whether *Cspg4*-lineage pericytes proliferate after MI, we treated *Cspg4^CreER/+^* *Rosa26^tdT/+^* mice with BrdU to label cells undergoing DNA replication (30). *Cspg4^CreER/+^* *Rosa26^tdT/+^* mice were given a single injection of BrdU immediately after surgery and then freely available water containing diluted BrdU during the time course of injury. BrdU was then detected in tdTomato^+^ cells using flow cytometry (Figure 4A). The percentage of proliferating tdTomato^+^ pericytes in hearts at post-MI days 2 and 4 was indistinguishable from that seen in sham control hearts (Figure 4B). However, the percentage of BrdU^+^ pericytes 7 days after MI significantly increased, suggesting increased proliferation of pericytes following the inflammatory window (Figure 4B). Although BrdU labels newly synthesized DNA of actively proliferating cells undergoing DNA replication, it does not accurately reveal the time course of cell proliferation. To quantify pericyte proliferation at the precise MI time point, a single injection of 5-ethynyl-2′-deoxyuridine (EdU) was administered 4 hours before the isolation of hearts (31) (Figure 4C). Complementing the BrdU proliferation results, EdU^+^ pericytes remained low at MI days 2 and 4, peaked at MI day 7, and declined between 7 and 14 days of injury (Figure 4D). We also observed an increased presence of tdTomato^+^ pericytes in the infarct regions, most significantly between post-MI days 7 and 14, corresponding with a marked reduction (percentage and number) of pericytes in the remote, noninjured areas (Figure 4, E–G). Together, our results show that *Cspg4*-lineage pericytes followed a temporal pattern of proliferation and accumulation around the area of injury during MI-induced cardiac remodeling.

### Single-cell RNA-Seq of Cspg4-lineage pericytes reveals enrichment of ECM genes.

**Figure 5:**
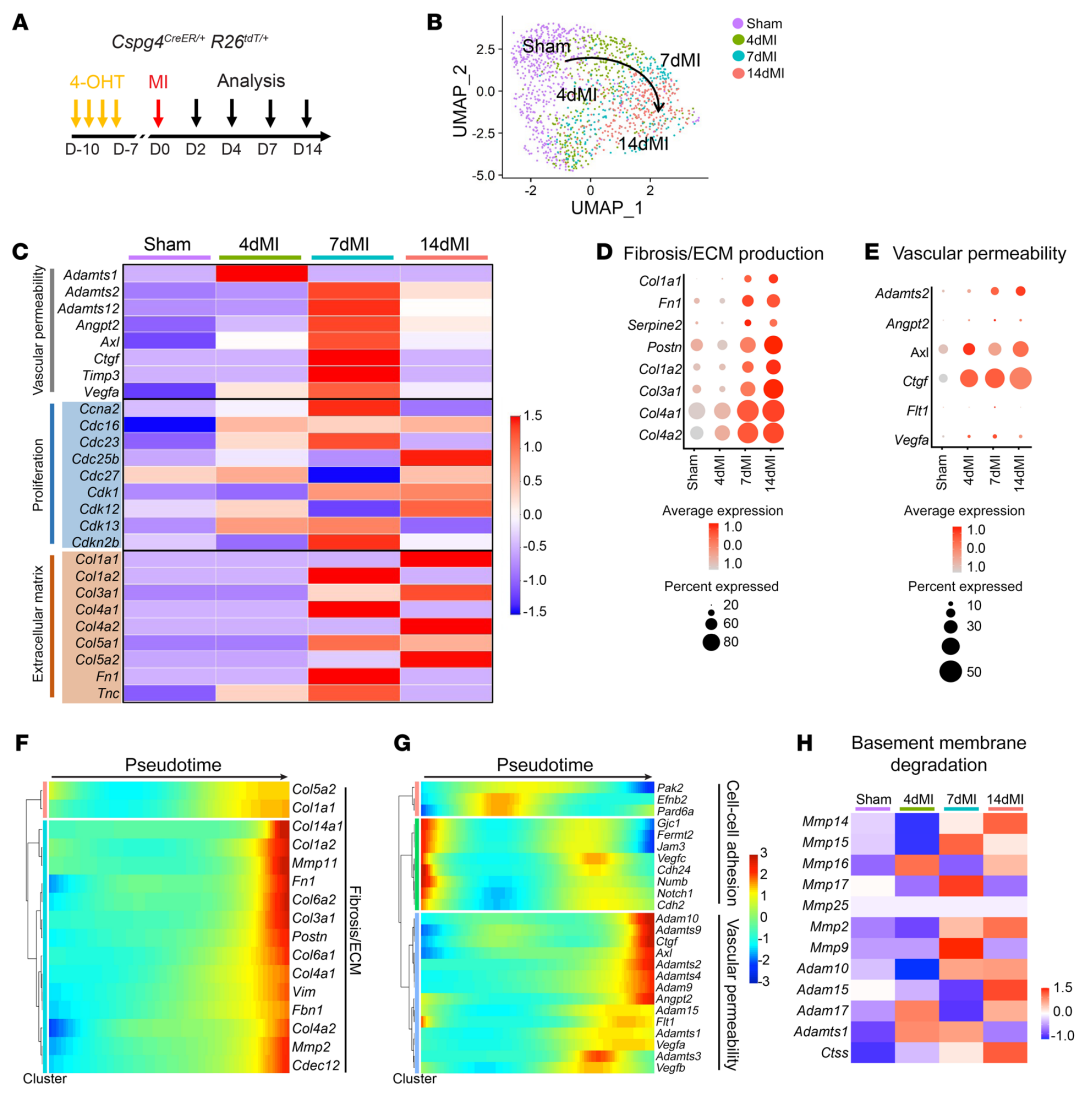
Single-cell transcriptional sequencing of cardiac pericytes reveals the induction of profibrotic gene signatures following MI. (**A**) Schematic for TAM injection before MI. Hearts were analyzed 4, 7, and 14 days after MI. (**B**) UMAP of single pericytes isolated from sham-operated hearts and after specified time points after MI. (**C**) Heatmap representation of genes associated with vascular permeability, cell proliferation, and ECM production in sham and MI isolated pericytes. The scale represents normalized expression. (**D** and **E**) Dot plots indicating the expression of selected marker genes associated with fibrosis and vascular permeability in sham and MI pericytes. The colored scale represents the average expression levels. The dot size represents the percentage of cells expressing the gene of interest. (**F** and **G**) Pseudotime expression of genes related to fibrosis and ECM, cell-cell adhesion, and vascular permeability in cardiac pericytes. The scale represents normalized expression. (**H**) Heatmap representation of genes associated with basement membrane degradation in sham and MI isolated pericytes. The scale represents normalized expression.

To elucidate the molecular mechanisms underlying the pericyte response to MI, we isolated pericytes from *Cspg4^CreER/+^* *Rosa26^tdT/+^* mice that had undergone sham or MI surgery after 4, 7, or 14 days to represent the inflammatory, proliferative/reparative, and maturation phases of ischemic remodeling, respectively, and performed scRNA-Seq via the 10x Genomics platform (Figure 5A and Supplemental Figure 5, A and B). Following quality control and filtering, we analyzed 5,311 cells and visualized cell clustering by uniform manifold approximation and projection (UMAP) (32) (Supplemental Figure 5, A and B). Clustering analysis revealed distinct populations acquired at each time point, with 1 significant pericyte population emerging as evidenced by the expression of *Cspg4*, *Mcam*, *Pdgfrb*, and *Notch3* (Supplemental Figure 5, B–D). Other minor clusters identified by our analysis corresponded to fibroblasts (*Pdgfra*, *Col1a1*, *S100a4*, *Ddr2*) (33), ECs (*Pecam1*, *Flt1*, *Icam2*, *Ly6a*, *Cdh5*) (34), immune cells *(Ptprc*, *Mrc1*, *Cd68*, *Cd14*) (35), and smooth muscle cells (SMCs) (*Acta2*, *Tagln*, *Myh11*) (36) (Supplemental Figure 5C). To specifically interrogate the pericyte response to MI, we focused our analysis on cells that expressed canonical mural cell markers but lacked expression of SMC genes (Supplemental Figure 5C). The extracted pericyte population was reclustered to visualize transcriptionally distinct cellular phenotypes following MI (Figure 5B). We analyzed differentially expressed genes in pericytes from post-MI days 4, 7, and 14 compared with pericytes from sham-operated hearts and conducted gene ontology (GO) analysis to determine the biological processes that were enriched. Pericytes, in response to ischemic cardiac injury, expressed an overabundance of genes related to vascular permeability, proliferation, ECM production, and fibrosis, which peaked at post-MI day 7, confirming their role in vascular stability, and suggesting that pericytes contributed to fibrotic scar formation (Figure 5, C–E). Notably, angiogenesis and cell motility genes were significantly enriched in pericytes 4 days after MI (Supplemental Figure 5F). By post-MI day 7, pericytes displayed increased expression of genes related to extracellular structure and cell substrate adhesion, indicating active engagement in ECM production and organization (Supplemental Figure 5F). Pericytes isolated at post-MI days 7 and 14 were also defined by GO terms related to translation and ribosome biogenesis, signifying increased protein production during the reparative and maturation phases of cardiac remodeling (Supplemental Figure 5F). We next sought to determine how fibrosis- and vascular integrity–related genes were altered in our single pericyte transcriptomics as they responded to ischemic injury by conducting pseudotime analysis using the Monocle algorithm (37). Pseudotime created a gene expression trajectory beginning with pericytes acquired from sham-treated mice and progressing through 4-, 7-, and 14-day MI (Supplemental Figure 5G). We then constructed an aggregated pseudotime transition of pericytes from sham to 4-, 7-, and 14-day MI for selected genes. We observed increased gene expression of markers related to fibrosis and vascular permeability from sham throughout the injured state (Figure 5, F and G, and Supplemental Figure 5, H and I). Conversely, cell-cell adhesion genes decreased in their dynamic expression pattern as pericytes transitioned from sham to the injured state (Figure 5G). Furthermore, we observed a relative upregulation of genes such as membrane type 1 matrix metalloproteinases (*Mmp14*, *Mmp15*, *Mmp17*), metalloproteinases (*Mmp2*, *Mmp9*), as well as other protease enzymes in injured pericytes as compared with sham (Figure 5H). In a separate experiment, basement degradation enzymes were upregulated in pericytes isolated from 2- and 4-day MI hearts, particularly *Ctss*, a protease involved in ECM degradation and advancement of lung pathogenesis (38) (Supplemental Figure 5J). The transcriptional profile of pericytes supports our experimental data on mural cell separation from the vasculature following MI and positioning at the site of injury 7 and 14 days after MI, which involves disruption and retraction from the basement membrane and subsequent vascular leakage.

### Pericytes of the Tbx18 lineage adopt a profibrotic phenotype following MI.

We next sought to validate our findings using a separate and independent mouse model to label pericytes. Recent studies have reported *Tbx18* as a gene marker for tracing cardiac and brain pericytes (5, 15). To validate that cardiac pericytes adopt a profibrotic phenotype, we lineage traced *Tbx18*-expressing pericytes by generating *Tbx18^CreER/+^* *Rosa26^tdT/+^* mice. We administered TAM before surgery to label *Tbx18*-expressing pericytes. We then explored gene expression profiles in sham-operated mice and in the peri-infarct and infarct regions 7 days after MI using scRNA-Seq (Supplemental Figure 6, A and B). We excluded cell contaminants (i.e., fibroblasts, cardiomyocytes, endothelial cells, SMCs, and immune cells), with the remaining *Tbx18*-expressing cells showing enriched expression of common pericyte genes, including *Rgs5*, *Mcam*, *Pdgfrb*, *Notch3*, and *Cspg4* (Supplemental Figure 6C). Unsupervised clustering of genes using pseudotime analysis revealed a transition from a quiescent to an activated state, consistent with our previous study of pericytes from the *Cspg4* lineage (Supplemental Figure 6, D and E). To establish overlap between lineage-tracing strategies, we evaluated the expression of *Cspg4* in the *Tbx18* lineage and *Tbx18* in the *Cspg4* lineage (Supplemental Figure 6F). We found that the *Cspg4*-lineage–tracing mouse model was more effective for labeling of pericytes than of ECs, fibroblasts, SMCs, or immune cells and compared with the *Tbx18*-lineage–tracing approach, which labeled a smaller percentage of pericytes (Supplemental Figure 6, G and H). Collectively, our scRNA-Seq data using 2 independent lineage-tracing strategies revealed that ischemic injury induced gene expression related to fibrosis and vascular permeability in cardiac pericytes.

### TGF-β signaling drives the profibrotic pericyte response to ischemic cardiac insult.

**Figure 6:**
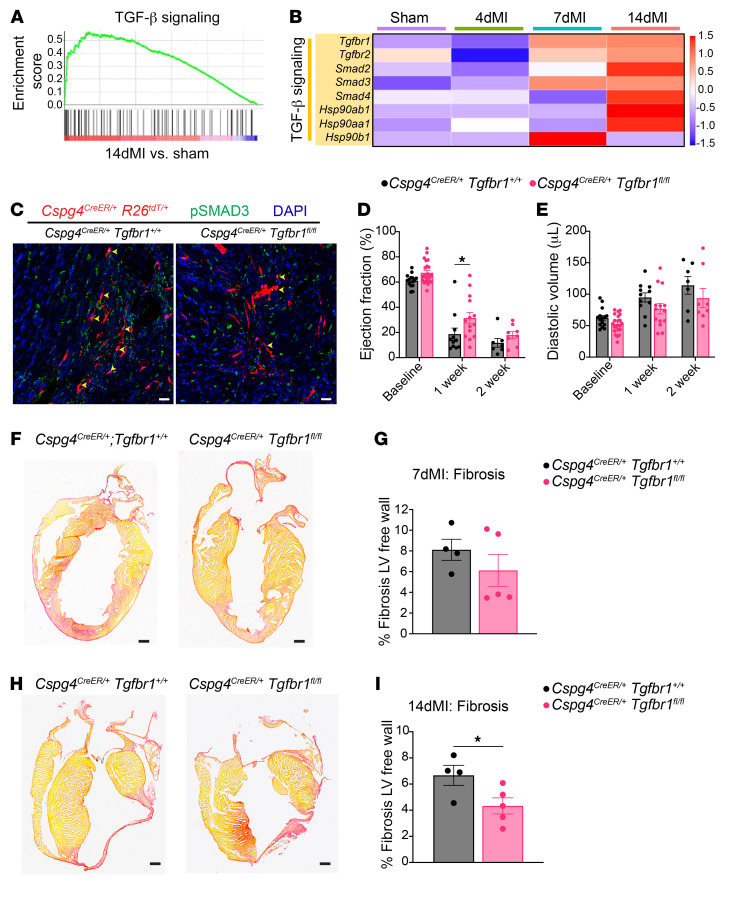
Deletion of *Tgfbr1* in cardiac pericytes. (**A**) GSEA of TGF-β signaling in pericytes isolated from hearts 14 days after sham or MI surgery. (**B**) Heatmap representation of genes related to TGF-β signaling in cardiac pericytes isolated from sham-operated hearts and hearts at early and late stages of MI. The scale represents normalized expression. (**C**) Immunohistochemical analysis of pericytes (red) and p-SMAD3 (green) in control and experimental mice 7 days after MI. Yellow arrowheads highlight p-SMAD3 in tdTomato^+^ pericytes. Scale bars: 20 μm. Images are representative of 3 control and 3 experimental hearts. Evaluation of (**D**) cardiac function through the measurement of ejection fraction and (**E**) cardiac morphometry by analysis of left ventricular diastolic volume in control *Cspg4^CreER/+^* *Tgfbr1^+/+^* and experimental *Cspg4^CreER/+^* *Tgfbr1^fl/fl^* mice. *n* = 15 control and *n* = 22 experimental mice at baseline; *n* = 11 control and *n* = 14 experimental mice 1 week after MI; *n* = 7 control and *n* = 8 experimental mice 2 weeks after MI. Data were analyzed by 2-way ANOVA with Šidák’s multiple-comparison test. Fibrosis in control and experimental mice (**F** and **G**) 7 days after MI and (**H** and **I**) 14 days after MI as measured by Picrosirius red staining. Collagen fibers, red. Live myocardium, yellow. *n* = 4 control and *n* = 5 experimental mice were analyzed by unpaired, 2-tailed Student’s *t* test. Scale bars: 500 μm. Data in **F** and **H** are representative of 4 control and 5 experimental hearts. *P < 0.05.

To identify pathways that correlate with the proremodeling pericyte phenotype, we performed gene set enrichment analysis (GSEA). We discovered that TGF-β signaling was highly enriched in pericytes after 14 days of MI (Figure 6A). Genes related to TGF-β signaling, such as *Tgfbr1*, *Tgfbr2*, *Smad2*, *Smad3*, and *Smad4*, were upregulated in 7- and 14-day MI pericytes (Figure 6B). We observed an increased abundance of phosphorylated-SMAD3 (p-SMAD3) in the nuclei of *Cspg4* lineage pericytes at 7- and 14-day MI, confirming the induction of TGF-β signaling (Supplemental Figure 7A). On the basis of these data, we speculated that TGF-β signaling might drive the cardiac pericyte injury response.

To determine the significance of TGF-β signaling in cardiac pericytes, we generated *Cspg4^CreER/+^* *Rosa26^tdT/+^ Col1a1^GFP/+^* *Tgfbr1^fl/fl^* mice (Supplemental Figure 7B), administered TAM to the mice for 4 days starting 1 week before sham or MI surgery, and analyzed hearts 1 and 2 weeks following MI when TGF-β signaling was most upregulated (Figure 6, A and B). In this transgenic mouse model, TAM administration resulted in the deletion of *Tgfbr1* in tdTomato^+^ pericytes. To investigate the impact of *Tgfbr1* deletion on pericyte accumulation in the left ventricle 14 days after MI, we isolated *Cspg4*-lineage pericytes using FACS. Although the percentage of tdTomato^+^ cells was unchanged between control and *Cspg4^CreER/+^* *Tgfbr1^fl/fl^* mice, the percentage of pericytes coexpressing tdTomato^+^ and *Col1a1-GFP* was modestly reduced (Supplemental Figure 7, C and D). Gene expression analysis showed a decrease in the expression of *Smad3* in tdTomato^+^ cells from *Cspg4^CreER/+^* *Tgfbr1^fl/fl^* mice, which was not observed in GFP^+^ cells, indicating a disruption in the canonical TGF-β1 signaling pathway specifically in pericytes (Supplemental Figure 7, E and F). Furthermore, we observed reduced expression of phosphorylated SMAD3 (p-SMAD3) in *Tgfbr1*-depleted pericytes compared with expression in controls at post-MI days 7 and 14, whereas ALK1 protein expression was unaltered between the 2 groups 7 days after MI (Figure 6C and Supplemental Figure 7, G and H). After subjecting mice to MI, we observed a modest improvement in ejection fraction on day 7 after MI following the deletion of *Tgfbr1* in the *Cspg4* lineage (Figure 6, D and E). However, cardiac function and diastolic volume were indistinguishable from controls 2 weeks after MI. Although the scar area was not significantly different between *Cspg4^CreER/+^* *Tgfbr1^fl/fl^* mice and *Cspg4^CreER/+^* *Tgfbr1^+/+^* control mice 7 days after MI, we did observe a significant decrease in scar area 14 days after MI (Figure 6, F–I). Vascular permeability based on dextran outside vessels in the BZ regions did not significantly differ between control and *Cspg4^CreER/+^* *Tgfbr1^fl/fl^* mice (Supplemental Figure 7I). Also, we did not observe a significant difference in cellular apoptosis, cardiomyocyte cross-sectional area, heart weight to body weight ratio (HW/BW), or vascular density when comparing control and experimental mouse strains following MI (Supplemental Figure 8, A–F). Additionally, cardiac ejection fraction, diastolic volume, and heart weight to tibia length (HW/TL) in mice subjected to sham surgery and analyzed up to 28 days after surgery were unchanged following *Tgfbr1* deletion in pericytes (Supplemental Figure 9, A–C). These observations suggest that TGF-β signaling contributed to pericyte activation 2 weeks after myocardial injury, consistent with a proremodeling phenotype that resulted from increased expression of basement degradation and vascular permeability genes.

### Deletion of fibronectin in cardiac pericytes does not alter cardiac physiology following MI.

Fibronectin (FN) has been shown to support EC-pericyte interactions that promote vascular basement membrane stability (39). Our previous cellular analysis and scRNA-Seq data identified *Fn1* as being among the most upregulated genes in pericytes after MI (Figure 6, C–F, and Supplemental Figure 3B), suggesting that FN may regulate the conversion of pericytes toward a profibrotic phenotype. To investigate the role of *Fn* in the pericyte response to ischemic injury, we generated *Cspg4^CreER/+^* *Rosa26^mTmG/+^* *Fn^fl/fl^* mice, which were administered TAM for 7 days followed by a 1-week washout period before MI surgery (Supplemental Figure 10A). Deletion of *Fn* in the *Cspg4* lineage did not significantly affect survival or cardiac function at day 14 after MI (Supplemental Figure 10, B and C). Although expression of FN protein was decreased in *Cspg4*-lineage pericytes following the genetic deletion of *Fn* compared with controls, fluorescently tagged pericytes (GFP^+^) showed continued coexpression of PDGFRβ in both control and experimental mice at post-MI day 14 (Supplemental Figure 10D). Additionally, the association of GFP^+^ pericytes with CD31^+^ ECs was not significantly altered following 7 days of MI in *Cspg4^CreER/+^* *Rosa26^mTmG/+^* *Fn^fl/fl^* mice compared with control mice (Supplemental Figure 10, E and F). Therefore, we concluded that FN expression in pericytes might not be critical for their response to ischemia and suggests that pericytes play a minor role in fibrosis formation.

### In vivo genetic ablation of pericytes increases vascular leakage following MI.

**Figure 7:**
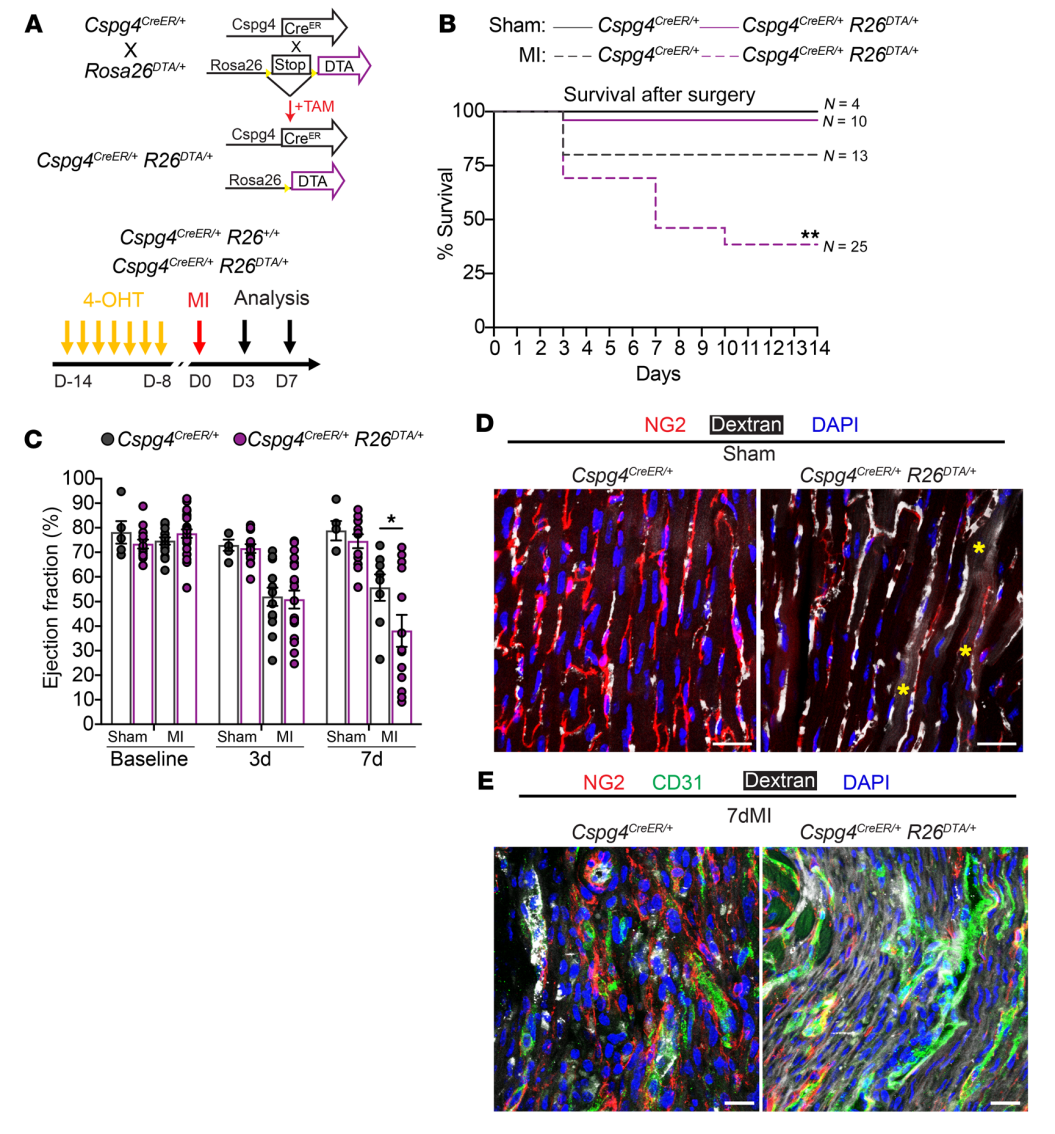
*Cspg4*-lineage pericytes are required for vascular stability after MI. (**A**) Schematic representation for the generation of *Cspg4^CreE2/+^* *R26^DTA/+^* mice before TAM administration and performance of sham or MI surgery. (**B**) Kaplan-Meier survival curve for control *Cspg4^CreER/+^* and experimental *Cspg4^CreERT2/+^* *R26^DTA/+^* mice following sham or MI surgery. *Cspg4^CreER/+^* sham, *n* = 4 and MI, *n* = 13; *Cspg4^CreERT2/+^* *R26^DTA/+^* sham, *n* = 10 and MI, *n* = 25. Survival curves were analyzed using a log-rank (Mantel-Cox) test. (**C**) The ejection fraction was measured in sham-operated and MI-injured animals, starting at baseline and up to 7 days after surgery. Baseline *Cspg4^CreER/+^* sham, *n* = 5 and MI, *n* = 15, 3dMI *Cspg4^CreER/+^* sham, *n* = 5 and MI, *n* = 13, 7dMI *Cspg4^CreER/+^* sham, *n* = 5 and MI, *n* = 8; baseline *Cspg4^CreERT2/+^* *R26^DTA/+^* sham, *n* = 13 and MI, *n* = 25, 3dMI *Cspg4^CreERT2/+^* *R26^DTA/+^* sham, *n* = 13 and MI, *n* = 19, 7dMI *Cspg4^CreERT2/+^* *R26^DTA/+^* sham, *n* = 12 and MI, *n* = 13. Data were analyzed by 2-way ANOVA with Šidák’s multiple-comparison test. (**D**) Immunohistochemical analysis of sham-operated hearts to visualize pericytes (NG2, red) and dextran-based permeability from the vasculature. (**E**) Immunohistochemical analysis of hearts 7 days after MI to visualize pericytes (NG2, red) and ECs (CD31, green) and dextran to assess vascular permeability. Scale bars: 20 μm (**D** and **E**). Images in **D** and **E** are representative of 3 control and 3 experimental hearts. *P < 0.05 and **P < 0.01.

We next sought to determine whether ablation of pericytes alters cardiac physiology at baseline or following MI. We generated compound heterozygous mice carrying the *Cspg4^CreER/+^* allele and the Rosa26–diphtheria toxin fragment A (DTA) allele (hereafter referred to as *Cspg4^CreER/+^* *R26^DTA/+^*) for an inducible Cre-dependent depletion of pericytes in vivo (40) (Figure 7A). TAM was administered to mice for 7 consecutive days, 2 weeks before MI surgery (Figure 7A). Mouse survival was not different between *Cspg4^CreER/+^*, *R26^DTA/+^*, and control mice following sham surgery. However, MI resulted in a significant decrease in survival of *Cspg4^CreER/+^* and *R26^DTA/+^* mice and a decline in cardiac ejection fraction in the remaining animals, coinciding with a loss of pericyte density (Figure 7, B and C, and Supplemental Figure 11A). To determine whether vascular permeability contributed to the decrease in cardiac function, we infused mice with dextran 7 days after surgery. In the sham-treated control mice, we observed dextran in the intact vasculature lined with NG2-expressing pericytes (Figure 7D). In contrast, we detected considerable dextran extravasation into the extravascular space even in the sham mice after pericyte ablation (Figure 7D). After MI, we observed substantial amounts of dextran outside of vascular beds in the infarct region of *Cspg4^CreER/+^* *R26^DTA/+^* mice compared with controls (Figure 7E and Supplemental Figure 11B). Further analysis 7 days after MI revealed no significant differences in cardiomyocyte hypertrophy or fibrosis when comparing control mice with pericyte-depleted mice (Supplemental Figure 11, C–F). Pericytes are critical for the formation of the blood-brain barrier (BBB) (41). To assess BBB integrity in our conditional mouse model, we injected Alexa Fluor 647–labeled BSA (BSA-647), which can cross the BBB as a result of vascular impairment (42). In both the presence of pericytes and DTA-mediated targeted ablation of pericytes, no leakage of BSA-647 was observed within the homeostasis regulatory structure, the hypothalamus (Supplemental Figure 11, G and H). Our data suggest a lack of BBB permeability due to the ablation of *Cspg4*^+^ cells following either sham or MI surgery, indicating that the decline in cardiac function and survival was associated with dysfunction in the myocardium. Therefore, our data show that pericytes are essential for maintaining vascular stability following acute MI and that their depletion compromises vascular integrity.

## Discussion

Pericytes are a mural cell population of the microvasculature required for vascular homeostasis and exhibit dynamic changes following ischemic injury in the heart, kidney, and brain, contributing to tissue remodeling (11, 43). Although a few studies have characterized cardiac mesenchymal cell and pericyte behavior during pathological damage (15, 16), the role of these cells in fibrotic and vascular remodeling has yet to be extensively described in the ischemic adult heart. Here, we used lineage reporters and 2 independent transgenic mouse lines to label cardiac pericytes and track their fate in response to myocardial injury. Our data demonstrate that the response of pericytes to ischemic cardiac insult was characterized by the temporal progression of (a) detachment and migration away from the microvasculature, (b) accumulation in the infarct and BZ via active proliferation, and (c) induction of genes encoding ECM molecules and basement degradation enzymes resulting in destabilization of the microvasculature and exacerbation of scar formation after MI. Using single-cell transcriptomics, we discovered that the TGF-β pathway was highly enriched in post-MI pericytes, and knockout of *Tgfbr1* in mural cells promoted a transient improvement in cardiac function and a reduction in cardiac fibrosis. Supporting the role of pericytes in maintaining cardiac homeostasis, pericyte-specific ablation in the heart increased vascular leakage and reduced cardiac function after MI, leading to considerable lethality. Our data are consistent with findings following pericyte deficiency in the brain, which increased neurovascular decoupling and formation of neuroinflammatory lesions leading to neuronal degeneration (44, 45). Understanding how pericytes respond to MI may accelerate the development of strategies to stimulate blood vessel growth into ischemic cardiac tissue and reduce scar formation.

While total pericyte depletion in the heart is pathogenic, it is interesting to speculate that a physiological reduction in the investment of pericytes among ischemic BZ blood vessels may stimulate vascular instability and potentially neovascularization. Pericytes and ECs communicate through mechanisms facilitated by intact basement membrane components such as collagen I/IV and fibronectin (46). Degradation of the basement membrane occurs through proteolytic processes leading to the release of proangiogenic factors that stimulate EC migration and sprouting (47). It is appealing to consider the involvement of pericytes in the breakdown of the endothelial basement membrane via the production of metalloproteinases during cardiac stress, as previously shown with the rapid production of MMP9 during brain ischemia (48). However, the expression of pericyte-specific proteases has yet to be identified in the heart. Here, we identified the enrichment of *Mmp2*, *Mmp9*, *Mmp14*, *Mmp15*, and *Mmp17* in 7- and 14-day MI pericytes. MMP-14 has been shown to remodel the ECM through degradation and is a critical factor in post-MI remodeling (49). Furthermore, basement membrane breakdown can induce neovascularization by permitting EC migration and survival (47). In addition to crosstalk with ECs, cardiomyocytes have been shown to communicate with pericytes through the secretion of pro–nerve growth factor, leading to pericyte detachment following MI (50). Therefore, it will be essential to investigate whether vascular instability induced by pericyte delamination represents a proangiogenic mechanism that can be modified and tested to improve perfusion of the ischemic heart.

Given the potential heterogeneity of pericytes in various disease states (3), we sought to investigate whether pericytes exhibit specialized functions in response to ischemic stress. Using scRNA-Seq, we observed that pericytes were responsive to MI through the upregulation of genes related to vascular permeability, proliferation, and ECM production beginning at post-MI day 7. In contrast, cardiac fibroblasts were activated much earlier, within 3–4 days of injury. Resident cardiac fibroblasts secrete large amounts of ECM 4–7 days after injury and lose their proliferative capacity by days 7–10 after MI (51). Our results demonstrate that pericytes have temporally and functionally distinct roles. For example, *Cspg4*-lineage pericytes showed peak proliferation and ECM secretion beginning 7 days after injury, and this “activated” phenotype persisted in the injured heart for 14 days. We also observed a similar induction of gene programs associated with ECM remodeling and the wound healing response following the analysis of pericytes acquired from the *Tbx18* lineage 7 days after MI, suggesting that pericytes may be a regulator of cardiac fibrosis. Future studies are needed to determine the significance of cardiac pericytes during the later stages of scar maturation and their potential re-recruitment to the vasculature. These findings can provide essential information for therapies to promote vascular stability and reduce pathologic accumulation of ECM.

scRNA-Seq also revealed a correlation between the profibrotic pericyte phenotype and TGF-β signaling. TGF-β signaling has been shown to regulate pericyte contractility (52), maturation (53), and conversion toward a profibrotic phenotype (54). Disrupting TGF-β signaling in *Cspg4*-lineage pericytes via genetic deletion of TGF-βR1 improved cardiac function 7 days after MI and showed reduced fibrosis 14 days after MI. However, this phenotype was transient. Since cardiac pericytes are shown to have concurrent roles in post-MI scar formation and vascular remodeling, interpretation of the results from *Tgfbr1* mice is difficult. Alternative signaling pathways induced by ischemia include PDGFB/PDGFRβ and JAGGED1/NOTCH3, leading to the deregulation of EC proliferation and vascular remodeling (6). In our study, we cannot exclude the possibility of concurrent mechanisms that may alter the pericyte response to myocardial injury.

Treatment and interventions for vascular diseases have primarily focused on restoring the perfusion of damaged tissue through the promotion of angiogenesis or coronary collateral formation, with little consideration of supportive pericytes that may mediate these processes. Recent studies show that pericytes act as critical sensors to control capillary flow (55) and provide electrometabolic support for cardiomyocytes (56). Isolated pericytes from cardiac, adventitial, and skeletal sources are reported to stimulate proangiogenic and antifibrotic effects facilitated through a unique secretome (57, 58). In the heart, transplantation of adventitial pericytes improved vascularization and decreased the incidence of fibrosis in ischemic large animal models (59). On the basis of our studies and others, we recognize that pericytes participate in physiological and pathological processes that require further investigation to define their roles in ECM and vascular remodeling.

## Methods

### Animal models

C57BL/6J mice were purchased from The Jackson Laboratory (stock number 000664). *Cspg4-dsRED*–transgenic reporter mice express red fluorescent protein under the control of the *Cspg4* promoter and enhancer elements, allowing the visualization of cardiac pericytes in situ and during FACS analysis (25). *Cspg4^CreERT2^* mice express the TAM-inducible Cre-recombinase under the control of *Cspg4* promoter and enhancer elements. *Cspg4^CreERT2^* mice, purchased from The Jackson Laboratory (stock no. 008538), were used for labeling of cardiac pericytes in the adult heart and validated to have *Cspg4*-expressing cells (60). *Rosa26^tdTomato^* and *Rosa26^mTmG/mTmG^* mice were used for fluorescence-based lineage tracing analysis and purchased from The Jackson Laboratory (stock nos. 007909 and 007576, respectively). *Rosa26^DTA^* mice expressed diphtheria toxin in Cre-expressing cells (40) and were used in our study for ablation of pericytes. *Rosa26^DTA^* mice were purchased from The Jackson Laboratory (stock no. 009669). *Col1a1^GFP^*-transgenic mice were used to track the expression of *Col1a1* in cardiac fibroblasts and pericytes and were purchased from the Jackson Laboratory (stock no. 013134). Deletion of *Fn* was accomplished using mice containing loxP sites flanking exon 1 of the *Fn1* gene purchased from the Jackson Laboratory (stock no. 029624). *Tgfbr1* deletion was performed using mice containing loxP sites flanking exon 3 of the *Tgfbr1* gene purchased from Jackson Laboratory (stock no. 028701). *Tbx18^CreERT2^* mice have the T-box18 promoter/enhancer that drives the expression of a TAM-inducible Cre recombinase and were used for labeling of cardiac pericytes. *Tbx18^CreERT2^* mice were purchased from The Jackson Laboratory (stock no. 031520).

### Myocardial infarction

Mice were anesthetized by exposure to an isoflurane-oxygen mixture, intubated with a 20 gauge angiocath, and ventilated with a volume-cycled rodent ventilator at 130 cycles/min (SAR-830, CWE Inc.). After the left thoracotomy between the fourth and fifth ribs, the pericardium was opened, and the left anterior descending coronary artery (LAD) was ligated intramurally 1–2 mm from the tip of the customarily positioned left atrium with an 8-0 suture. Lungs were reinflated, and the chest was closed in 2 layers; the ribs (inner layer) were closed with 6-0 coated vinyl sutures in an interrupted pattern. The skin was closed using 6-0 nylon or silk sutures in a subcuticular manner. Ventilation was maintained until sufficient spontaneous breathing occurred, and the mice were allowed to recover in a temperature-controlled chamber until they resumed full alertness and mobility. For the localized injection of TAM to label and trace cardiac pericytes after MI injury, 2 μL 4-hydroxytamoxifen (MilliporeSigma) was injected 1 mm above the ligation site following MI.

### Echocardiography

Echocardiography was performed using the Vevo 2100 (Fuji Film Visual Sonics) at baseline and over a longitudinal course to evaluate cardiac function and recovery from surgical procedures. Briefly, animals were lightly anesthetized to ensure similar heart rates across animals and to allow for comparison between animals and groups. Hair was removed from the chest, and the mouse was gently restrained and placed on a micromanipulator platform following electrophysiologic and myocardial structure-function analysis. Vevo LAB software (Fuji Film Visual Sonics) was used to acquire cardiac function and physiological measurements.

### Isolation of cardiac pericytes by FACS for gene expression analyses

Mice were injected with heparin (MilliporeSigma) and euthanized 20 minutes later (see data in Figure 1B, Figure 2, F–I, and Supplemental Figure 2C). The hearts were dissected and cannulated for perfusion with 30 mL PBS. The hearts were then perfused with 5 mL collagenase type II solution diluted in PBS (Worthington Biochemical Corporation) and cut into small pieces. Minced myocardial tissue was collected and placed in 10 mL collagenase solution on a rotator at 37°C for 1 hour with periodic pipetting to ensure complete digestion. Digested cells were centrifuged through a 40 μm filter (350*g* for 10 min) to collect cells. Cellular debris was removed using a Debris Removal Kit (Miltenyi Biotec), and cells were resuspended in FACS buffer (1× PBS, 1 mM EDTA, 25 nM HEPES, 1%FBS). If antibody staining was necessary, resuspended cells were incubated with antibodies for 30 minutes on ice. To stain for biotinylated antibodies, a streptavidin–APC–eFluor 780–conjugated (Supplemental Table 1) secondary antibody was added for 30 minutes on ice after washing with FACS buffer. Cells were analyzed or sorted using a BD FACSAria II cell sorter. All flow cytometric data were analyzed using FlowJo software, version 10.

### Langendorff technique for the isolation of cardiac pericytes by FACS for gene expression analyses, flow cytometry, and ImageStream analysis

To begin the procedure (see data in Figure 1G, Figure 3, B and C, Supplemental Figure 3, C and D, Supplemental Figure 6, C–F, and Supplemental Figure 7, E and F), mice were anesthetized using a ketamine-xylazine solution, and the chest was opened to reveal the aortic arch. A small incision was made high up in the aortic arch, and a 22 gauge cannula (Radnoti) was inserted. After aortic cannulation, collagenase type II (Worthington Biochemical) dissolved in perfusion buffer (potassium chloride solution) was perfused through the heart at 37°C by suspension of the aorta on a Radnoti EZ Myocyte/Langendorff Isolated Heart System (Radnoti) for 8 minutes. Upon completion of digestion, the heart was immersed in a perfusion buffer supplemented with 0.5% FBS, and the heart was further dissected manually. Next, cells were allowed to settle to the bottom of a 15 mL conical tube to eliminate cardiomyocytes by placing the tube in a 37°C bath for 15 minutes. The supernatant was then passed through a 100 μm filter and centrifuged (10 min, 600*g*, 4°C). The supernatant was aspirated, and the pellet was resuspended in 100 μL FACS buffer (0.5% BSA in PBS) and incubated with flow cytometric antibodies at the appropriate dilution for 20 minutes on ice. Cells were washed once with FACS buffer and centrifuged (10 min, 600*g*, 4°C) before placing them in the appropriate volume and tube for downstream analysis. Cells were analyzed and sorted using a BD FACSAria II cell sorter or an Amnis ImageStream GenX. Flow cytometric data were analyzed using FlowJo software, and ImageStream data was analyzed using IDEAS software. Antibodies used for flow cytometry, FACS, and Image Stream experiments are listed in Supplemental Table 1.

### RNA isolation, cDNA synthesis, and RT-qPCR

To perform gene expression analyses, isolated cells were sorted directly into TRIzol LS Reagent (Thermo Fisher Scientific). RNA was extracted from sorted cells or whole heart samples following the manufacturer’s protocol. Extracted RNA was quantified using a NanoDrop Spectrophotometer (Thermo Fisher Scientific) and then converted into cDNA using the iScript cDNA Synthesis Kit (Bio-Rad) following the manufacturer’s instructions. RT-qPCR reactions were prepared with SYBR Green Master Mix (Bio-Rad), and specific primers designed for each target gene are listed in Supplemental Table 2. The reactions were run on a CFX96 Touch Real-Time PCR Detection System (Bio-Rad), and analysis was performed using the ΔΔCt method.

### Administration of dyes to visualize heart vasculature

Before sacrificing the animals, the mice were administered isolectin GS-IB4 (0.25 μg/mouse, Thermo Fisher Scientific) and dextran biotin 70,000 MW (25 mg/mouse, Thermo Fisher Scientific) via tail vein injections, and the mice were allowed to rest for at least 1 hour before harvesting of tissue.

### Immunofluorescence staining (frozen)

Mice were injected with heparin (MilliporeSigma) and euthanized after 20 minutes, followed by perfusion with 30 mL PBS and then fixation in 4% PFA for 2 hours at 4°C. After a quick wash in PBS, the hearts were stored in 30% sucrose overnight at 4°C. The hearts were then embedded in an OCT (Thermo Fisher Scientific) and maintained at –80°C. The blocks were sectioned using a Leica cryostat at a thickness of 8–100 μm. The sections were mounted on Colorfrost Plus microscope slides (Fisher Scientific) and stored at –20°C until imaging.

For IF staining, slides with 8 μm sections were left at room temperature for 10 minutes, washed 3 times with PBS, and permeabilized with 0.25% Triton X-100 (prepared in PBS) for 10 minutes. Slides were treated with a blocking buffer (10% normal goat serum [NGS], PBS–0.1%Tween 20) for 1 hour at room temperature, followed by incubation with primary antibodies (diluted in blocking buffer) overnight at 4°C in the dark. The next day, slides were washed 3 times with PBS–0.1% Tween 20 and incubated with secondary antibodies diluted in a blocking buffer for 1 hour at room temperature. After another 3 washes with PBS–0.1% Tween 20, coverslips were mounted onto the slides using a mounting medium containing DAPI (Vector Laboratories). The slides were imaged with a Zeiss confocal microscope (LSM880 or LSM700), and image processing was done using ZEN 2 (blue edition) software.

### Immunofluorescence staining (paraffin)

To perform IF staining of paraffin-embedded sections, slides were deparaffinized in a series of xylenes, followed by 3-minute incubations in 100% ethanol (EtOH) (3 times) and 95% EtOH (1 time), and were then placed in distilled water. Specifically, for IF, antigen retrieval was performed in pH 6 Dako Target Antigen Retrieval buffer (Agilent Technologies), followed by quenching in 3% H_2_O_2_ in 15 mM NaCl/100 mM Tris pH 7.5 (TN buffer). Slides were then blocked in Blocking Reagent (PerkinElmer) diluted to 0.5% in 1X TN. Primary antibodies were incubated overnight at 4°C at validated dilutions. After overnight incubation (16–18 hours), slides were washed in 1X TN buffer, followed by secondary antibody incubation for 2 hours at room temperature. Amplification was performed as necessary (biotin-streptavidin or HRP-tyramide). Slides were washed with 1X TN following secondary incubation, with the final wash containing DAPI (Thermo Fisher Scientific) for at least 10 minutes to stain for nuclei. Slides were mounted with VECTASHIELD Anti-Face Mounting Media (Vector Laboratories) before being imaged on an Olympus Confocal Microscope IX81. Antibodies and dilutions for IF (frozen, paraffin, and vascular dyes) are listed in Supplemental Table 3.

### Assessment of proliferation using BrdU and EdU

To label proliferating cells in the heart, mice were injected with 100 μg BrdU (Roche) immediately after sham or MI surgery. Following the initial injection, mice were administered water containing BrdU (1 mg/mL) to label proliferating cells continuously, and the water was changed every other day. BrdU labeling was then analyzed by flow cytometry using the APC BrdU Flow Kit (BD Pharmingen). For EdU experiments, 1 mg EdU (prepared in DMSO and diluted in saline, Thermo Fisher Scientific) was injected intraperitoneally, and mice were sacrificed 4 hours later. Hearts were processed for IF staining as described in the previous section. The Click-it (Plus) EdU Imaging Assay (Thermo Fisher Scientific) was used for fluorescence marking of EdU in cells following the manufacturer’s instructions.

### Single library preparation and processing of single pericytes

#### Single-cell quality control and library preparation of Cspg4-lineage pericytes.

Cells were sorted from *Cspg4^CreER/+^* *Rosa26^tdT/+^* hearts that had undergone the sham procedure or MI (4, 7, and 14 days; *n* = 2 hearts per sample), as described above, and were placed in DMEM without calcium (Gibco, Thermo Fisher Scientific) and 10% FBS. Next, the cell suspension was centrifuged, and all media except for roughly 40 μL were removed. The number of cells was counted using the TC20 Automated Cell Counter (Bio-Rad), and cell viability was assessed by trypan blue staining. Only cell populations exhibiting more than 80% viability were used. All cells were loaded to maximize the number of single cells acquired using the Chromium Single Cell 3′ Reagent Kit (10x Genomics). Libraries were prepared according to the manufacturer’s instructions using the Chromium Single Cell 3′ Library and Gel Bead Kit, version 3 (10x Genomics). Libraries were sequenced on a NovaSeq Sequencer (Illumina), with an average of 150,000,000 million reads sequenced per sample.

#### Single-cell quality control and library preparation of Tbx18-lineage pericytes.

Hearts from *Tbx18^CreER/+^* *Rosa26^tdT/+^* mice were extracted after sham surgery or 7 days after MI and were sorted for tdTomato expression via FACS. RNA libraries were prepared for sequencing according to the manufacturer’s instructions using the Chromium Single Cell 3′ Library and Gel Bead Kit, version 3 (10x Genomics). Sequencing was performed using an Illumina NovaSeq 6000 Sequencer. CellRanger (version 3.0.2) mkfastq was used to demultiplex the base call files, and CellRanger counts with an mm10 reference genome were used to align reads and UMI counts and generate filtered feature barcode matrices. Seurat (version 3) was used to process the data. Cells were filtered with minimum features of greater than 500 and minimum cells of greater than 3. Integration was performed using the IntegrateData() function, log normalization was performed, and the data were scaled using 2,000 variable features. Cells were clustered at a resolution of 0.5, and clusters were labeled according to the expression of various cardiac cell-type marker genes. Pseudotime analysis using processed data from Seurat was performed as described above.

#### Preliminary processing of raw sequencing reads.

CellRanger (version 2.2.0), was used to demultiplex each capture, process base-call files to fastq format, and perform 3′ gene counting for each cell barcode with the mouse reference data set (mm10, version 2.1.0).

#### Cell filtering and cell-type annotation and clustering analysis.

Quality control, identification of variable genes, principal component analysis, and nonlinear reduction using UMAP were performed using Seurat (version 3.0.0.9000) and R (version 3.5.1) for each time point separately. The integration function RunCCA was used to identify cell-type–specific clusters. Cell-type annotations were identified on the basis of significant cluster-specific marker genes and according to the Mouse Gene Atlas using Enrichr (enrichR_2.1). Cell-cycle scoring was performed, and variation was introduced as the number of genes involved in mitochondrial transcription, and cell-cycle phases S and G_2_/M were regressed during data scaling. UMAP-visualized data and cluster data were defined using a resolution of 0.5.

#### Pseudotime analysis.

Processed data from the Seurat object were extracted and analyzed in Monocle (version 2.10.0). The function estimateSizeFactors was used to normalize the mRNA expression between different cells, and estimateDispersions was used to estimate the dispersion of genes between cells. The detectGenes function filters genes expressed in 10 or more cells. The differentialGeneTest function was used to identify differentially expressed genes across the different conditions that could be used to order the cells. Dimensionality reduction was performed via the DDRTree method, and the orderCells function was used to learn a trajectory. A differentialGeneTest was performed on the data set to identify differentially expressed genes over pseudotime, and the plot_pseudotime_heatmap function was used to generate the pseudotime heatmap of individual genes.

### Data availability

The single RNA-Seq data that support this work are available under the NCBI Gene Expression Omnibus (GEO) accession codes GSE178469 (*Tbx18*-lineage) and GSE201947 (*Cspg4*-lineage).

### Code availability

All analyses were performed using standard protocols with previously described R packages (32).

### Statistics

All data are presented as the mean ± SEM. Statistical analyses were performed using an unpaired, 2-tailed Student’s *t* test for comparison of 2 groups and a 1- or 2-way ANOVA for comparison of multiple groups. Tukey’s post test was used to correct multiple comparisons made by 1-way ANOVA, and Šidák’s post test was used to correct multiple comparisons made by 2-way ANOVA. All measurements in this work were acquired from different biological samples, and no samples were measured more than once. Bar graph data were analyzed using GraphPad Prism 8.4.2 for Mac OS (GraphPad Software). A *P* value of less than 0.05 was considered statistically significant.

### Study approval

All animal studies, including husbandry, breeding, and experimental procedures, followed protocols approved by the University Committee on Animal Resources at the University of Rochester and the UCLA.

## Author contributions

PQ and SP designed the experiments, analyzed data, and drafted the manuscript. PQ and SP generated mouse models, performed injections, measured cardiac function, and isolated tissues and cells for experiments, including cell, gene expression, and histological analyses with assistance from PZ, KSSK, DW, KDS, KF, AP, LW, AD, MDT, EMR, KNBV, MLGH, and TTDP. PQ is listed first as co-author, as she was responsible for the primary handling of the manuscript, including the coordination and completion of the experiments necessary for the first submission of the manuscript and revisions. Specifically, PZ, KF, AP, LW, and AD assisted in generating animal models, conducted microsurgical techniques, and performed the analysis of physiological data under the supervision of RA. KSSK performed bioinformatics analysis of *Tbx18*- and *Cspg4*-lineage pericytes and conducted gene expression and IF analysis of *Cspg4*-transgenic mice under the supervision of RA. DW, KDS, and MDT conducted flow cytometric analysis and capture of pericytes to conduct qRT-PCR, analyzed cardiac function, and isolated tissue from *Tgfbr1* and DTA mouse models for histological examination under the supervision of PQ. KNBV and MLGH conducted flow cytometric and histological analysis of pericytes in *Cspg4* and FN mouse strains under the supervision of EMS and CJL. TTDP and EMR conducted a research of vascular perfusion in the brain of *Cspg4* and DTA mice following MI under the supervision of STC. EMS and RA conceived of the study, supervised the research, and cowrote the manuscript with input from all authors.

## Supplementary Material

Supplemental data

Supplemental video 1

Supplemental video 2

## Version 1 05/15/2023

Electronic publication
