# Supplementary material for: Cardiac pericytes mediate the remodeling response to myocardial infarction
Source: J Clin Invest. 2023 May 15;133(10):e162188. doi: 10.1172/JCI162188 (PMC10178847; doi:10.1172/JCI162188)
Supplement: Supplemental data [file jci-133-162188-s154.pdf]

Supplemental Material for:

Cardiac pericytes mediate the remodeling response to myocardial infarction

Pearl Quijada, Shuin Park, Peng Zhao, Kamal S.S. Kolluri, David Wong, Kevin D. Shih, Kai Fang, Arash Pezhouman, Lingjun Wang, Ali Daraei, Matthew D. Tran, Elle M. Rathbun, Kimberly N. Burgos Villar, Maria L. Garcia-Hernandez, Thanh T.D. Pham, Charles J. Lowenstein, M. Luisa Iruela-Arispe, S. Thomas Carmichael, Eric M. Small, Reza Ardehali

This file contains the following:
Supplemental Methods
Supplemental References
Supplemental Tables 1-3
Supplemental Figures 1-11

Supplemental Methods

Animal Models and Breeding

Mouse breeding and tamoxifen administration for individual experiments were: 1) *Cspg4*$^{dsRED/+}$ reporter mice were maintained as a single transgenic mouse strain or crossed to *Col1a1*$^{GFP/+}$ mice. 2) The breeding strategy to lineage trace cardiac pericytes: *Cspg4*$^{CreER/+}$ mice were crossed to either *R26*$^{tdTomato/tdTomato}$ or *R26*$^{mTmG/mTmG}$. Additionally, *Cspg4*$^{CreER/+}$; *R26*$^{tdT/+}$ were crossed to *Col1a1*$^{GFP/+}$. *Tbx18*$^{CreER/+}$ were crossed to *R26*$^{tdTomato/tdTomato}$. 3) The breeding strategy for deletion of *Tgfbr1* in cardia pericytes: *Cspg4*$^{CreER/+}$; *R26*$^{tdT/+}$; *Tgfbr1*$^{fl/+}$;*Col1a1*$^{GFP/+}$ mice were crossed to *R26*$^{tdT/+}$; *Tgfbr1*$^{fl/+}$; *Col1a1*$^{GFP/+}$mice. 4) The breeding strategy for deletion of *Fn1* in cardiac pericyte: *Cspg4*$^{CreER/+}$; *R26*$^{mTmG/+}$; *Fn*$^{fl/+}$ mice were crossed to *R26*$^{mTmG/+}$; *FN*$^{fl/+}$; *Col1a1*$^{GFP/+}$mice. 5) The breeding strategy for ablation of *Cspg4* expressing pericytes: *Cspg4*$^{CreER/+}$ mice were crossed to *R26*$^{DTA/+}$ mice. To induce Cre recombinase, transgenic mice were administered tamoxifen at a 100mg/kg dose for 4-7 days, with the last dose given at least one week before myocardial infarction surgery. Mice were maintained as mutant mice for no more than five generations before performing two backcrosses. Mice of the female and male sex were included in this study and aged between 12-16 weeks.

Picrosirius red staining

Frozen slides were stained with Picrosirius Red Stain Kit (Polysciences, Inc.). To begin, slides were removed from the -20°C freezer and allowed to thaw for 30 seconds. The sections were then hydrated with distilled water for 10 seconds, and Picrosirius red stain was added to the slides for 20 minutes. Following the 20-minute incubation, Picro Sirius red stain was rinsed with hydrochloric acid (HCl) for 1 minute twice. Following the second HCl wash, slides were placed in 70% Histology-grade ethanol for 3 minutes, followed by 100% Histology-grade ethanol for 3 minutes. Following ethanol incubation, slides were dried for 30 seconds before applying Permount and a cover slip. Stained slides were imaged by bright field imaging, and tile scanned at a magnification of 20x using the Applied Imaging Leica Aperio Verso scanner. Quantitation of Picro Sirius was performed using Image J Version 2.0. software (National Institutes of Health, USA) by normalizing the area of the Picro Sirius stain to the size of the left ventricular free wall.

In situ cell death detection

To detect apoptotic cells following myocardial infarction, sections of formalin-fixed and frozen embedded heart tissue were subjected to an in-situ cell death detection kit, fluorescein (Millipore Sigma). Briefly, tissue sections were thawed for 5 minutes and hydrated with PBS for 5 minutes. Slides were then subjected to a permeabilization solution (0.1% Triton-X-100, 0.1% sodium citrate) for 2 minutes. TUNEL reaction mixture was created and placed on slides for 1 hour at 37°C in a humidified atmosphere in the dark. Slides were washed twice with PBS and incubated with DAPI before coverslipping and imaging using confocal microscopy.

Measurement of cardiomyocyte size

Cardiomyocyte cross-sectional size was visualized in the border zone area of the left ventricular free wall following MI by staining with fluorescently labeled wheat germ

agglutinin. The quantification of cardiomyocyte size was performed with a semiautomated measurement of cell size using Imaris software, as previously reported (1).

Vascular permeability assessment in the brain

To assess vascular permeability in the brain following pericyte ablation, we administered Bovine serum albumin (BSA) conjugated to Alexa-Fluor 647 (BSA-AF647, 66.6kDa) in 50µl sterile saline per mouse via retro-orbital injection. To allow time for the dye to leak from the blood-brain barrier (BBB), mice were returned to their home cage to regain consciousness before euthanasia. We then proceeded to immunofluorescence staining on frozen brain sections. First, mice subjected to sham and MI surgeries were euthanized by isoflurane induction and decapitation one hour after a retro-orbital injection of BSA-AF647. Brains were isolated and fixed for 24 hours in 4% PFA at 4°C, then transferred to 30% sucrose for 2 days at 4°C before being flash-frozen in powdered dry ice and stored at -80°C. Coronal sections were cut at a thickness of 40µm using a cryostat (Leica CM3050) and slide-mounted on triple gelatin subbed Superfrost slides. Slides with mounted sections were lined with a hydrophobic barrier for immunofluorescence staining. The sections were washed 3 times with PBS, pH 7.4, incubated at 95°C in sodium citrate, pH 6.0, for antigen retrieval, rewashed 3 times with PBS, and permeabilized with 3 washes of 0.3% TritonX-100 in PBS (PBST). Sections were treated with 5% normal donkey serum (NDS) in PBST for 2 hours at room temperature, followed by incubation with a rat primary antibody to CD31 to detect endothelial cells in 2% NDS in PBST at 4°C for two days. After 3 washes with PBST, sections were incubated in 2% NDS in PBST with secondary antibody for 2 hours at room temperature, then washed 3 times in PBS and passed through a graded ethanol (50%, 70%, 90%, 100%, 100%, 1min each) and xylenes series (2 times, 3min each), and coverslipped with DPX mountant.

The posterior part of the anterior hypothalamic area was imaged using confocal laser scanning microscopy on a Nikon Eclipse Ti microscope using a 60x oil immersion objective and 40µm z-stack depth with a 0.5µm step size. Images were then processed in ImarisViewer.

Supplemental References

1. Gilda JE, Ko JH, Elfassy AY, Tropp N, Parnis A, Ayalon B, et al. A semiautomated measurement of muscle fiber size using the Imaris software. *Am J Physiol Cell Physiol.* 2021;321(3):C615-C31.

Supplemental Tables

Antibody	Manufacturer (catalog no.)	Dilution	Secondary	Manufacturer (catalog no.)	Dilution
CD146-APC	BioLegend (134712) Clone (ME-9F1)	1:100	NA		
CD31-APC	BioLegend (102410) Clone (390)	1:100	NA		
CD31-BV421	BD Biosciences (563356) Clone (390)	1:50	NA		
DRAQ5	Thermo Fisher Scientific (62251)	1:10,000	NA		
PDGFRβ-Biotin	Miltenyi Biotec (130-109-866) Clone (REA634)	1:10	Streptavidin, APC-eFluor 780	eBioscience (47-4317-82)	1:200

Supplemental Table 1. List of antibodies used for flow cytometric analysis.

Gene	Forward Primer	Reverse Primer
18s	CATGGCCTCAGTTCCGAAAA	CGAGCCGCCTGGATACC
Col1a1	AATGGCACGGCTGTGTGCGA	AACGGGTCCCCTTGGGCCTT
Col1a2	GCAGGTTCACCTACTCTGTCCT	CTTGCCCCATTCATTTGTCT
Col3a1	TCCCCTGGAATCTGTGAATC	TGAGTCGAATTGGGGAGAAT
Col3a1	TAGGACTGACCAAGGTGGCT	GGAACCTGGTTTCTTCTCACC
Cspg4	GCTGTCTGTTGACGGAGTGTT	CGGCTGATTCCCTTCAGGTAAG
Cspg4	TCTTACCTTGGCCTTGTTGG	ATGTGGAGAACTGGAGCAGC
Ctss	CTGGCCACGCTGCCATAAGA	TTTTCCCAGATGAGACGCCG
Fn	ACCCGTTTTCATCCAACAAGAG	CGGTATCCAGACACCACACTATCA
Fn	AGACCTGGGAAAAGCCCTACCAA	ACTGAAGCAGGTTTCCTCGGTTGT
Gapdh	AGGTCGGTGTGAACGGATTTG	TGTAGACCATGTAGTTGAGGTCA
Mcam	CCCAAACTGGTGTGCGTCTT	GGAAAATCAGTATCTGCCTCTCC
Mmp9	CTCTCCTGGCTTTCGGCTG	AGCGGTACAAGTATGCCTCTGC
Mmp17	CCGTTCCTCAGATGCCCAC	ACACCGTACAACTGCCAGAC
Notch3	AGTGCCGATCTGGTACAACTT	CACTACGGGGTTCTCACACA
Pdgfrb	TTCCAGGAGTGATACCAGCTT	AGGGGGCGTGATGACTAGG
Pdgfrb	GGGAGACACTGGGGAATACTTTTG	TGAACAGGTCCTCGGAGTCCATAG
Postn	AAGCTGCGGCAAGACAAG	TCAAATCTGCAGCTTCAAGG
Smad3	CCAGGCTGACATGGGCAAATGAAA	TGTCACAGTTTGCTGTGGCAATCC

Supplemental Table 2. List of primer sequences for qPCR.

Target Protein/Conjugated Labeling Dye	Manufacturer (catalog no.)	Dilution/Dose
ALK1	Novus Biologicals (NBP1-30982), Clone (94)	1:50
CD146	Abcam (ab75769), Clone (EPR3208)	1:100
CD31	Abcam (ab28364), Polyclonal	1:100
CD31	eBioscience (14-0311-82), Clone (390)	1:50
CD31	BD Biosciences (557355), Clone (MEC 13.3)	1:10
CD45	eBioscience (14-0451-82), Clone (30-F11)	1:50
CNN1	Atlas Antibodies (HPA014263), Polyclonal	1:100
ERG	Abcam (ab92513), Clone (EPR3864)	1:100
FN	Proteintech (15613-1-AP), Polyclonal	1:100
NG2	Millipore Sigma (AB5320), Polyclonal	1:50
NOTCH3	ABClonal (A3115), Clone A3115	1:50
p-SMAD3	ThermoFisher Scientific (44-246G), Polyclonal	1:100
PDGFRα	R&D Systems (AF1062), Polyclonal	1:100
PDGFRβ	R&D systems (AF1042), Polyclonal	1:200
PDGFRβ	Abcam (ab32570), Clone (Y92)	1:100
PDGFRβ	eBioscience (14-1402-82), Clone (APB5)	1:50
POSTN	R&D Systems (AF2955), Polyclonal	1:100
a-actinin (sarcomeric)	Sigma-Aldrich (A7811), Clone EA-53	1:400
Actin, a-smooth muscle	Sigma-Aldrich (A2547), Clone 1A4	1:100
Dextran Biotin 70,000 MW	Fisher Scientific (D1957)	25mg/mouse
In situ Cell Death Detection Kit, Fluorescein	Millipore Sigma (11684795910)	per manufacturer's instruction
Isolectin GS-IB4 (488 and 647)	Fisher Scientific (488-I21411 and 647-I32450)	0.25μg/mouse
Wheat Germ Agglutinin (647)	Fisher Scientific (W32466)	10μg/mL
Bovine Serum Albumin (647)	Fisher Scientific (A34785)	0.2mg/mouse

Supplemental Table 3. List of antibodies used for immunofluorescence.

Supplemental Figure 1

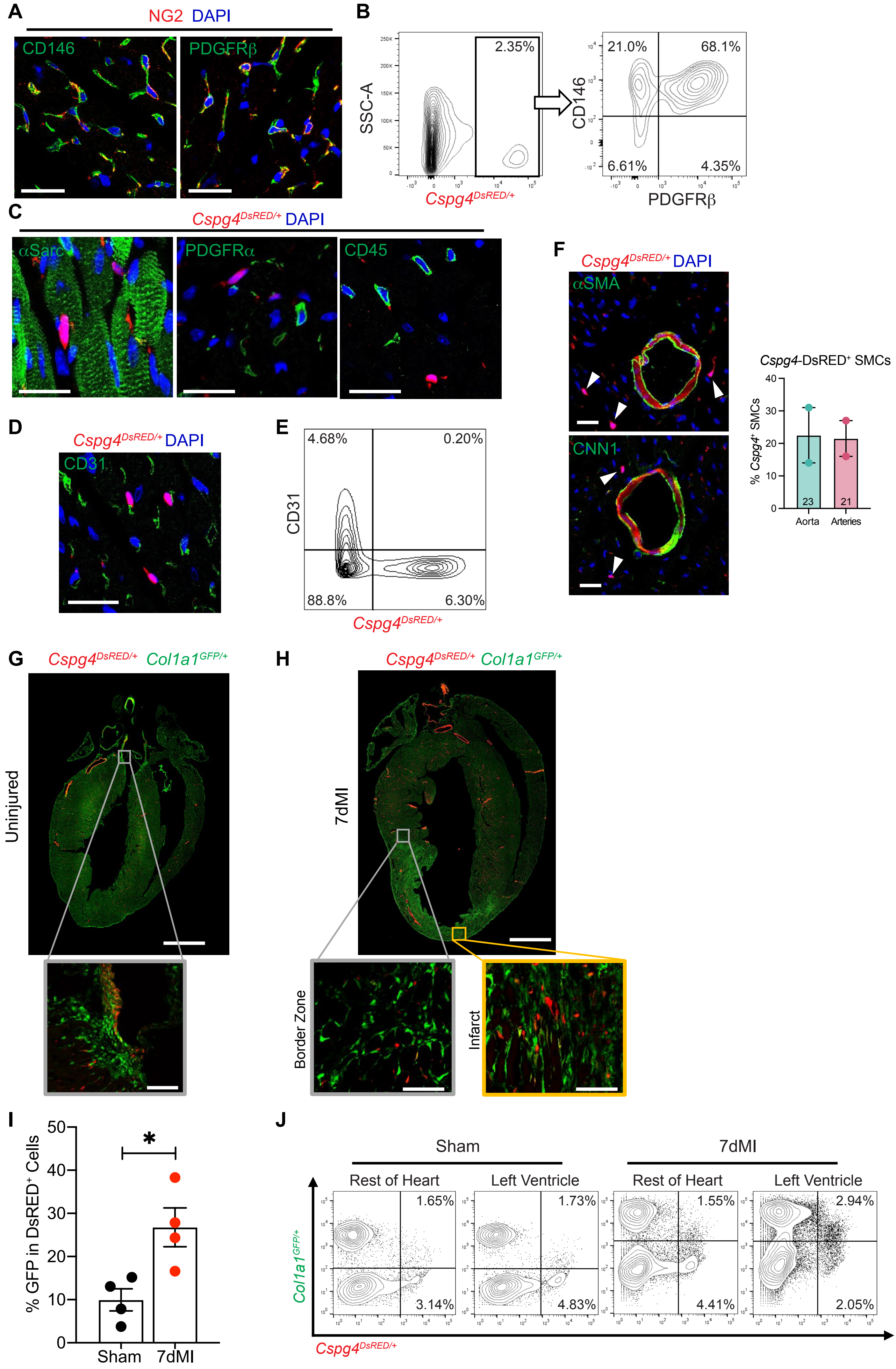

Supplemental Figure 1. *Cspg4*$^{DsRed/+}$ mouse strain labels cardiac pericytes. **(A)** Endogenous NG2-expressing cells co-express pericyte markers CD146 and PDGFRβ. **(B)** DsRed^{+} cells in *Cspg4*$^{DsRed/+}$ hearts express pericyte markers CD146 and PDGFRβ as determined by flow cytometric analysis. **(C)** The *Cspg4*$^{DsRed/+}$ pericyte reporter does not label cardiomyocytes, fibroblasts, or hematopoietic cells (markers in green). **(D)** DsRed^{+} cells are closely associated with CD31^{+} endothelial cells (green) but do not co-express CD31 based on immunohistochemical analysis. **(E)** DsRed^{+} cardiac pericytes do not express the vascular protein marker CD31 based on flow cytometric analysis. **(F)** Smooth muscle cells around larger vessels are labeled with *Cspg4*$^{DsRed/+}$ and can be differentiated from Cspg4^{+} pericytes, which reside as single cells in the interstitial area (arrowheads). (Right) Quantitation of *Cspg4*-dsRed cells that are also smooth muscle cell positive. N=2 hearts. Visualization of *Col1a1-GFP* expressing pericytes (DsRED^{+}) in the **(G)** Uninjured or **(H)** 7dMI heart. **(I)** Evaluation of GFP expression in the DsRed population of *Cspg4*$^{DsRed/+}$; *Col1a1*$^{GFP/+}$ hearts. Analysis was performed on hearts subjected to sham and 7dMI. N=4 for each group and analyzed using an unpaired two-tailed t-test. **(J)** Flow cytometric plots showing expression of DsRed and GFP following 7 days of MI. The distribution of cell populations was demonstrated in the left ventricle relative to the rest of the heart. Scale bar: 20μm in panels A, C, D, and F. Scale bar: 100μm in whole heart view and 50μm in insets shown in panels G and H. A, C, D, G, and H, Representative of three experiments. F, Representative of two experiments. *$p<0.05$.

Supplemental Figure 2

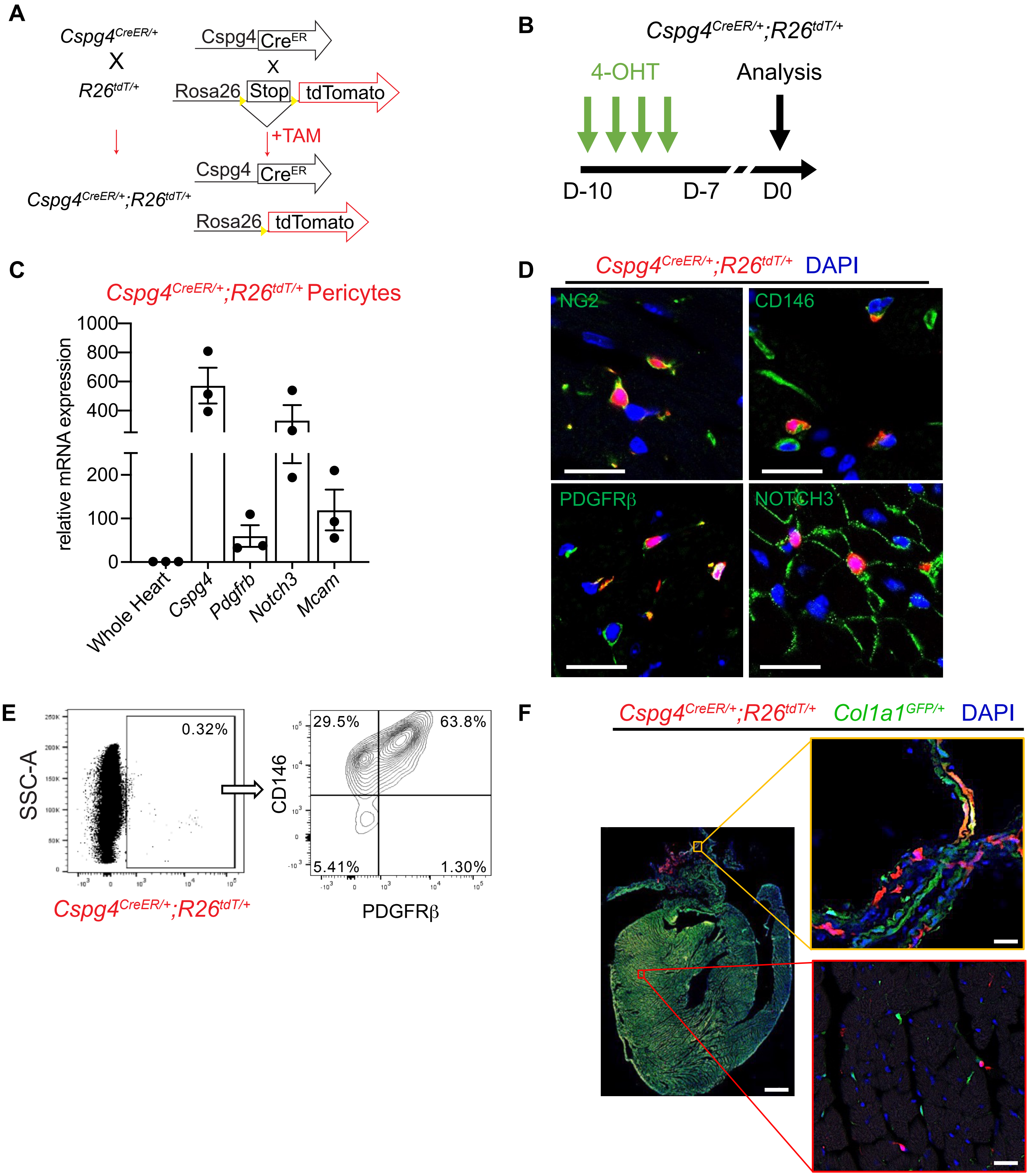

Supplemental Figure 2. ***Cspg4*$^{CreER/+}$*; Rosa26*$^{TdT/+}$ labels pericytes in the heart. (A)** Breeding schematic to generate *Cspg4*$^{CreER/+}$*; Rosa26*$^{tdT/+}$ double transgenic mice. **(B)** Schematic of tamoxifen administration of *Cspg4*$^{CreER/+}$*; Rosa26*$^{tdT/+}$ mice, followed by an analysis of uninjured hearts. **(C)** Gene expression of gene markers related to pericyte identity after isolation of *Cspg4*$^{CreER/+}$*; Rosa26*$^{tdT/+}$ cells using fluorescence-activated cell sorting. Relative gene expression was compared to the whole heart. N=3. **(D)** *Cspg4* lineage-traced tdTomato^{+} cells (red) express NG2 protein and other pericyte markers such as CD146, PDGFRβ, and Notch 3 (green). **(E)** tdTomato^{+} cells co-express CD146 and PDGFRβ as confirmed by flow cytometric analysis. **(F)** Expression of Col1a1-GFP is primarily observed in areas around the atria but not in the interstitial area of *Cspg4*$^{CreER/+}$*; Rosa26*$^{tdT/+}$*; Col1a1*$^{GFP/+}$ mice. Scale bar: 20μm in panels D. Scale bar: 50μm in whole heart view and 20μm in insets in panels F. D and F, Representative of three experiments.

Supplemental Figure 3

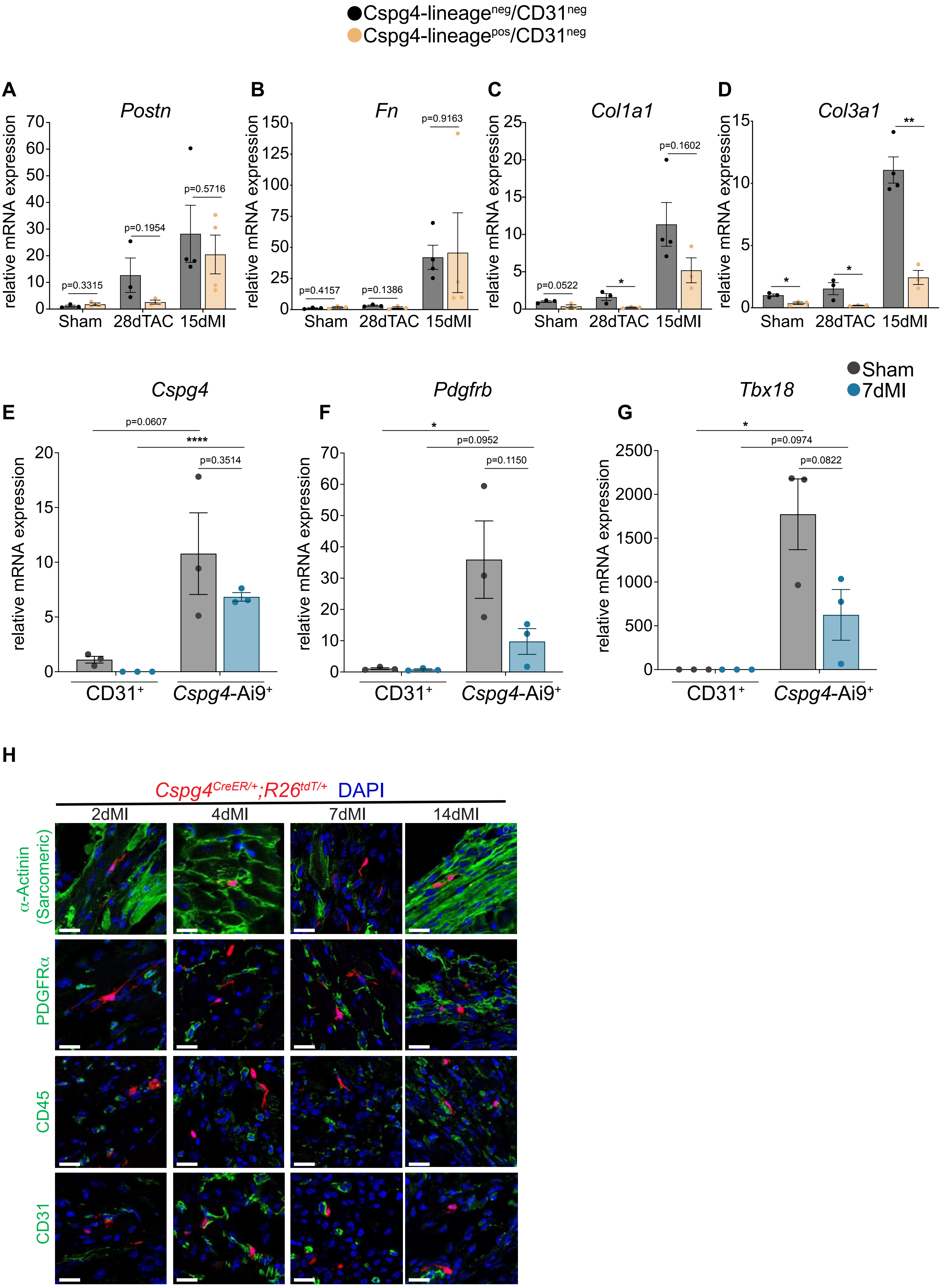

Supplemental Figure 3. Gene expression analysis of *Cspg4*-lineage pericytes compared to non-pericytes following pressure overload and myocardial infarction surgeries. Gene expression of **(A)** *Postn*, **(B)** *Fn*, **(C)** *Col1a1*, and **(D)** *Col3a1* collected from hearts subjected to sham surgery, 28 days of trans-aortic constriction (28dTAC) surgery or 15 days of myocardial infarction (15dMI). Expression was compared between *Cspg4*-lineage positive/CD31 negative cells (pericytes) and *Cspg4*-lineage negative/CD31 negative cells (fibroblasts/mesenchymal cells). Sham N=3 *Cspg4*-lineage negative and positive cells, 28dTAC N=3 *Cspg4*-lineage negative and positive cells, 15dMI N=4 *Cspg4*-lineage negative cells, and N=3 *Cspg4*-positive cells. Analysis was performed between *Cspg4*-lineage negative and positive cells under each condition using an unpaired two-tailed t-test. Gene expression of **(E)** *Cspg4*, **(F)** *Pdgfrb*, and **(G)** *Tbx18* in *Cspg4*-lineage pericytes as compared to CD31 positive endothelial cells. N=3 for all samples/conditions. Analysis was performed using One-Way ANOVA with Tukey multiple comparisons test. **(H)** Evaluation of tdTomato$^+$ cells and co-expression with markers of cardiomyocytes (α-sarcomeric actinin), fibroblasts (PDGFRα), hematopoietic (CD45), and endothelial cells (CD31) all represented in green and during a time-course of MI. Scale bar: 20µm. Representative of three experiments.

Supplemental Figure 4

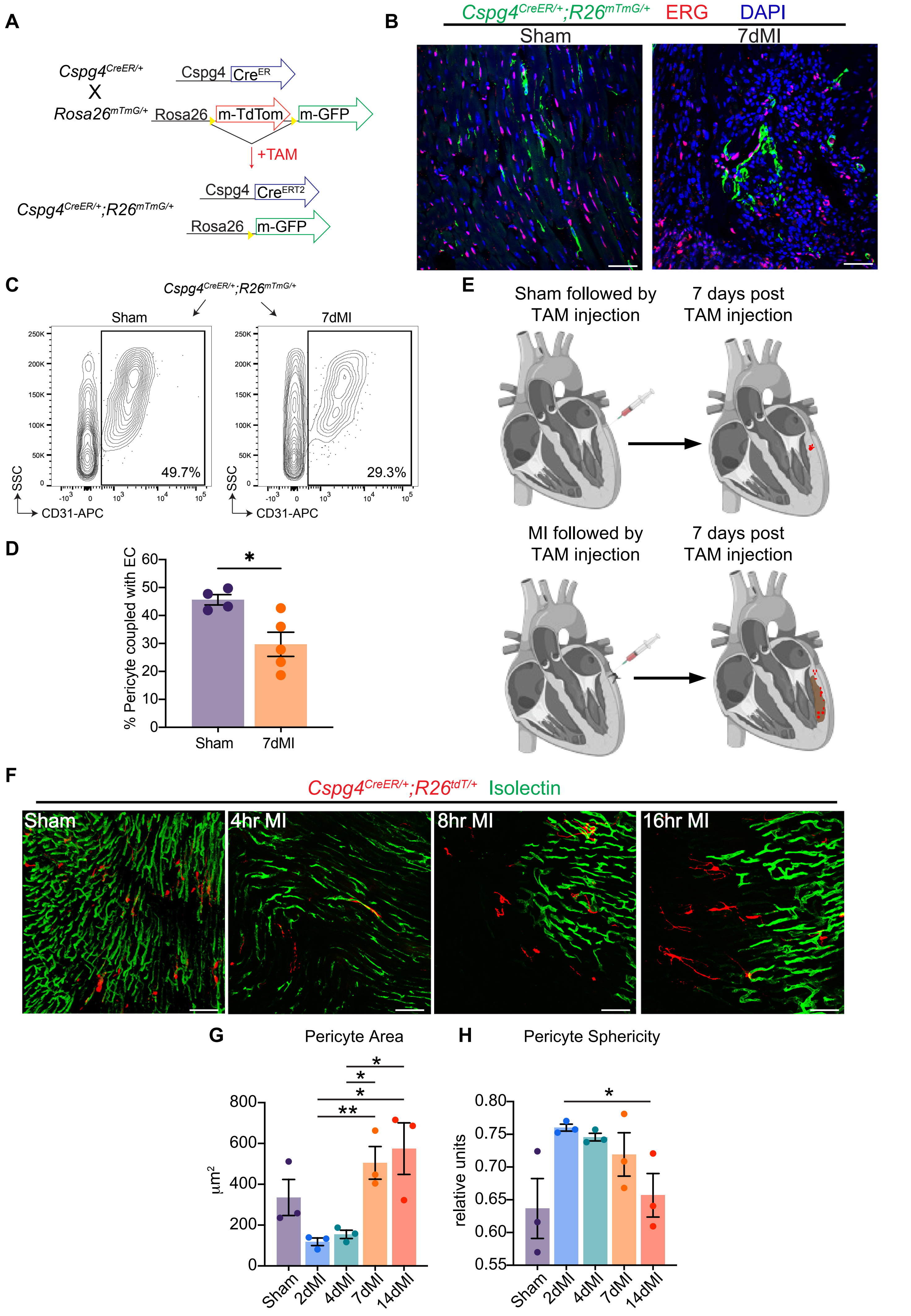

Supplemental Figure 4. *Cspg4*-lineage cells dissociate from the microvasculature after MI. (A) Schematic for generating *Cspg4*$^{CreERT2/+}$*; R26*$^{mTmG/+}$ mice before performing myocardial infarction (MI). **(B)** Immunohistochemical analysis of cardiac pericytes (green) in sham or 7d MI hearts. Vascular cells are labeled with endothelial cell-specific nuclear marker ERG (red). Scale bar: 40µm. **(C)** Analysis of pericyte association with CD31$^+$ endothelial cells in sham and 7-day MI hearts using flow cytometric analysis with quantitation **(D)** on the right. N=4 for both Sham and 7dMI samples analyzed using an unpaired two-tailed t-test. **(E)** Schematic representation of tamoxifen (TAM) injection in sham or MI hearts to observe localized expression of *Cspg4*-lineage pericytes 7 days post-injury. **(F)** Pericyte location relative to isolectin-labeled vasculature in healthy hearts (Sham) or following early time points post-MI (4, 8, and 16 hours). Scale bar: 50µm**.** Analysis of **(G)** Pericyte area (µm^2) and **(H)** Sphericity (relative units) in hearts subjected to sham or a time course of MI. N=3 samples per time-point. Data were analyzed by One-Way ANOVA with Tukey multiple comparisons test. B and F, Representative of three experiments. *$p<0.05$, **$p<0.01$.

Supplemental Figure 5

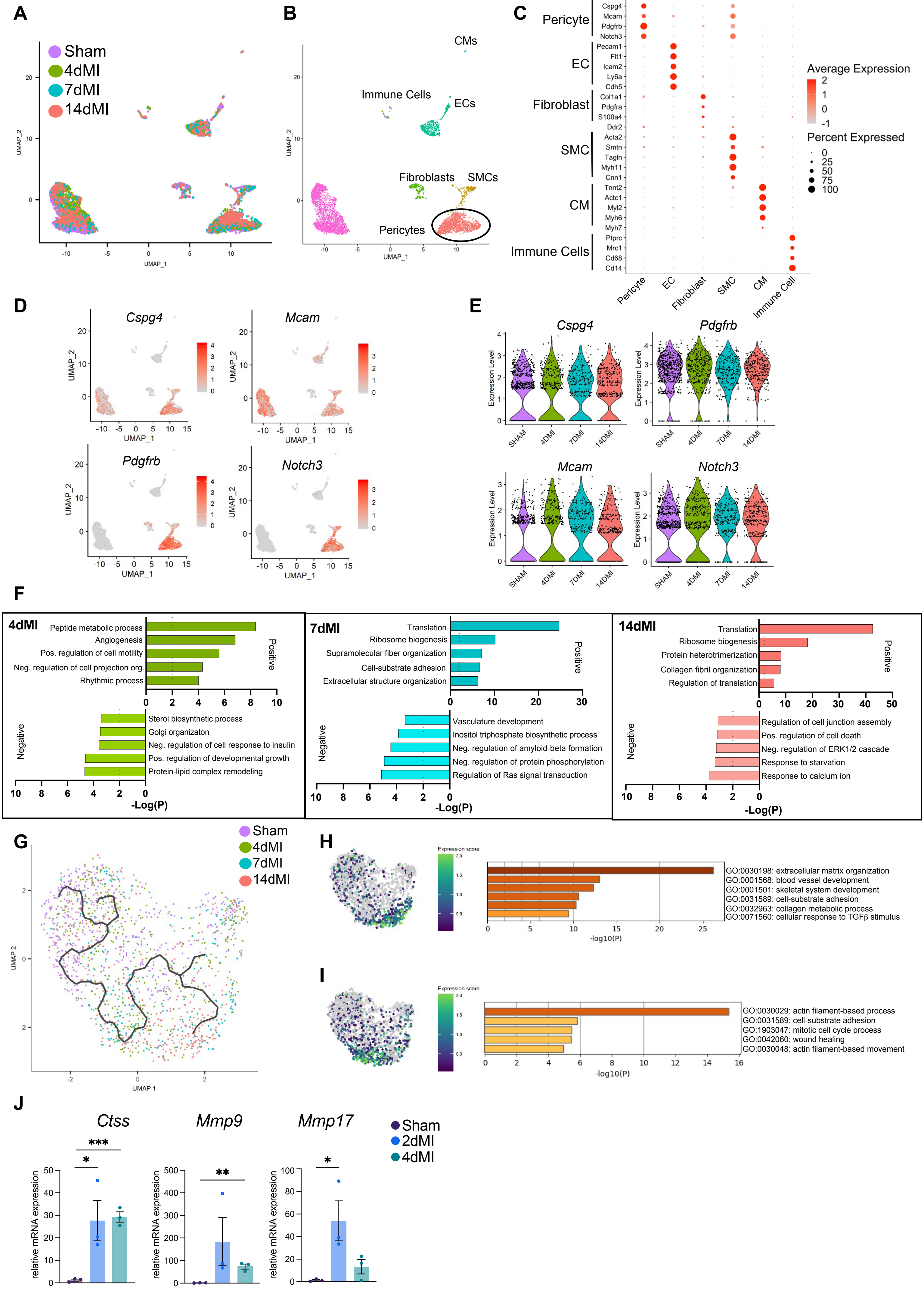

Supplemental Figure 5. Single-cell RNA-sequencing of *Cspg4*-lineage traced cardiac pericytes. (A) UMAP plot of cardiac cells collected for single-cell RNA-seq (scRNA-seq) following sham surgery or a time course of MI. **(B and C)** Annotation of cardiac cell types such as cardiomyocytes (CMs), immune cells, endothelial cells (ECs), fibroblasts, smooth muscle cells (SMCs), and pericytes was facilitated by the expression-specific cell markers as shown on the dot expression plot. The colored scale represents average expression, and circles represent the percent of cells expressed. **(D)** Expression of pericyte markers across the UMAP dictates a major cluster defined as pericytes, circled in **(B)**. **(E)** Expression of pericyte genes does not change significantly following MI. **(F)** Gene Ontology analysis of upregulated and downregulated genes at 4, 7, and 14 days after injury compared to cells acquired from sham hearts. **(G-I)** Pseudotime heat map analysis of pericytes isolated from Sham and MI hearts and gene ontology terms associated with cells from 7-day and 14-day MI. **(J)** Gene expression of motility genes in sham, 2- and 4-day MI pericytes. N=3 samples per time-point. Data were analyzed by One-Way ANOVA with Tukey multiple comparisons test. *$p<0.05$, **$p<0.01$, ***$p<0.001$.

Supplemental Figure 6

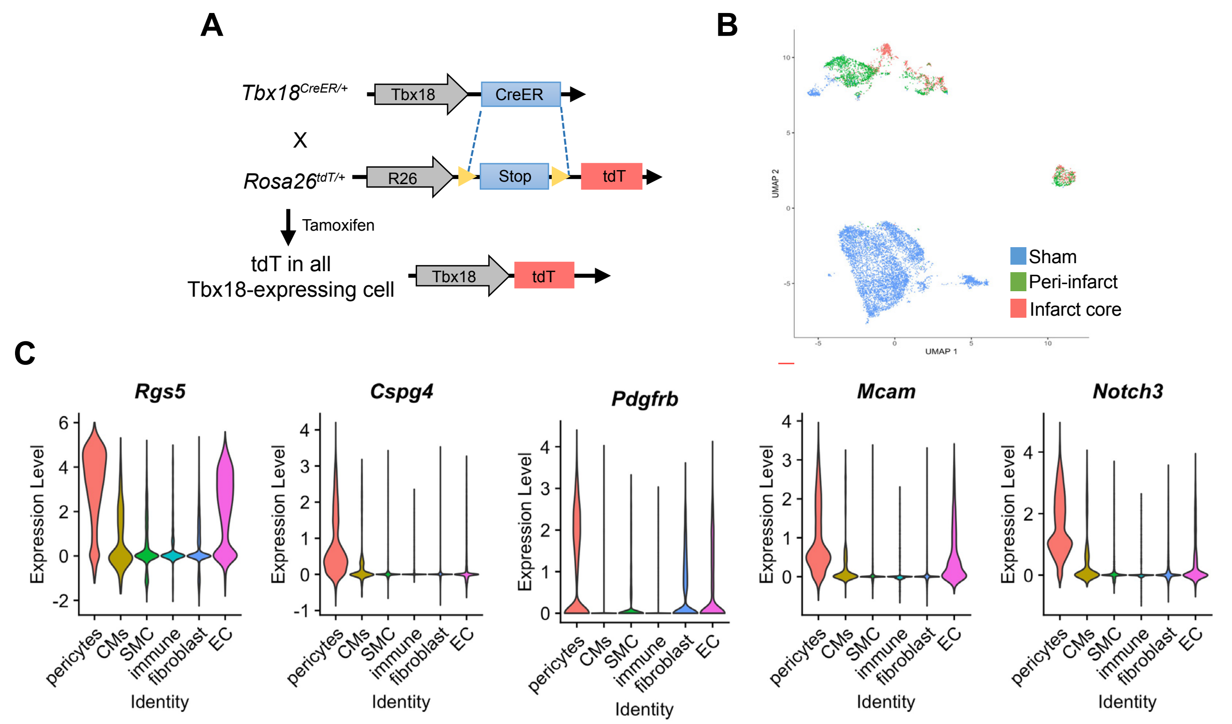

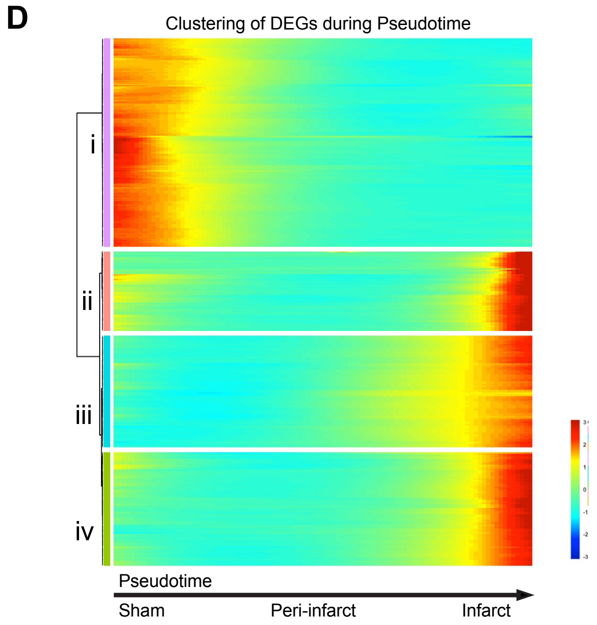

E

Highlighted GO Terms for Gene Group i-iv

Group	GO Categories	-log10
i	Blood vessel morphogenesis	12.6
i	Tight junction assembly	4.0
i	Cell-cell adhesion	3.9
ii	Extracellular matrix organization	9.8
ii	Wound healing	5.7
ii	Collagen catabolic process	2.8
iii	Extracellular matrix organization	6.7
iii	Epithelial cell proliferation	2.8
iii	Actin-mediated cell contraction	2.3
iv	Cellular response to reactive oxygen species	4.6
iv	Positive regulation of hydrolase activity	3.7
iv	Fibroblast proliferation	2.5

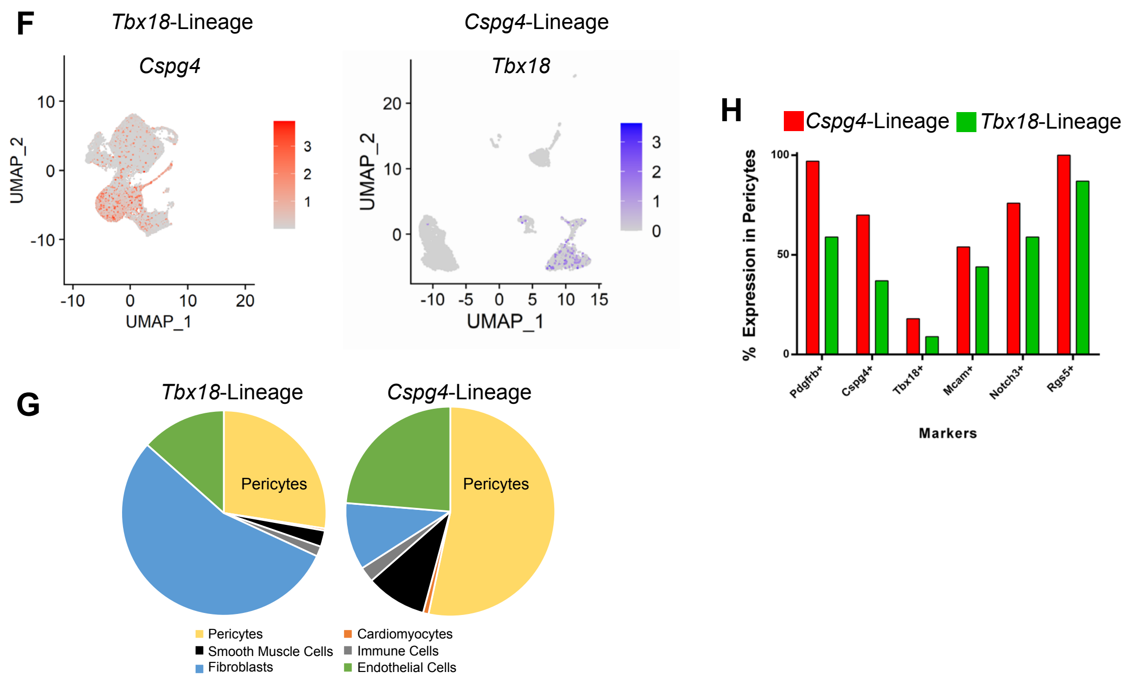

Supplemental Figure 6. Single-cell RNA-sequencing of *Tbx18*-lineage traced cardiac pericytes. **(A)** Schematic for the generation of fluorescently labeled *Tbx18*-lineage pericytes. **(B)** UMAP plot of pericytes acquired from sham and either the peri-infarct or infarct core of 7-day MI hearts. **(C)** Violin plots depicting canonical pericyte gene expression in the pericytes and other cell lineages. **(D)** Pseudotime heat plot of *Tbx18*-lineage cells from sham to the infarct region of 7-day MI hearts. **(E)** Highlighted Gene Ontology terms for genes located in the pseudotime heat map. Group i represents genes highly expressed in the sham, whereas Groups ii-iv represent genes highly expressed in the infarct pericytes. **(F)** *Cspg4* in cells acquired from the *Tbx18*$^{CreER/+}$ mouse strain (left) and *Tbx18* in cells acquired from the *Cspg4*$^{CreER/+}$ mouse strain (right). **(G)** Cell fractions in the *Tbx18* (left) or *Cspg4* (right) lineage. **(H)** The percentage of cells expressing canonical markers of pericytes (*Pdgfrb*, *Cspg4*, *Tbx18*, *Mcam*, *Notch3*, *Rgs5*) in both the *Cspg4* and *Tbx18* lineages.

Supplemental Figure 7

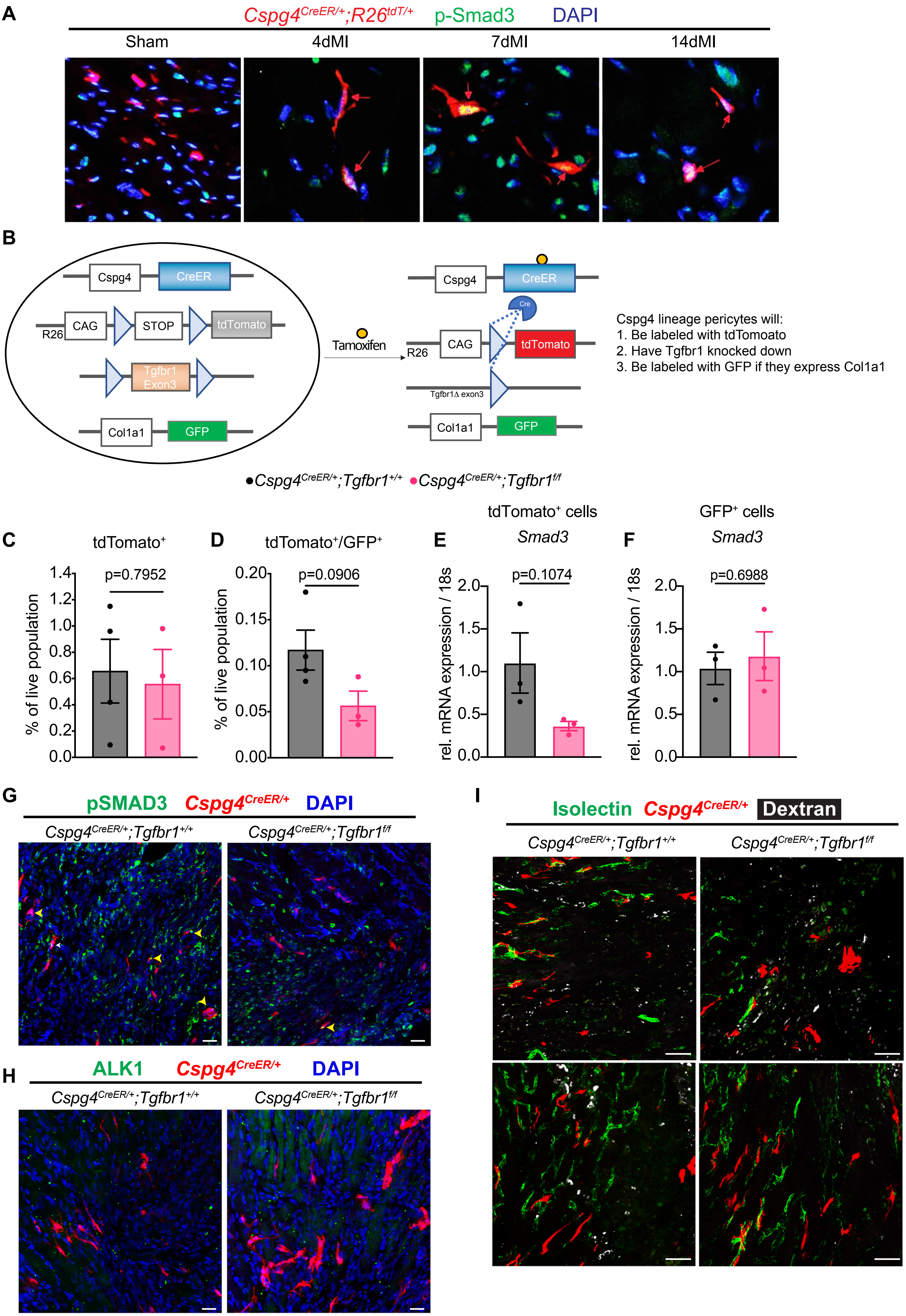

Supplemental Figure 7. Analysis of *Cspg4*-lineage cells following *Tgfbr1* deletion and after acute myocardial infarction. (A) Analysis of phosphorylated SMAD3 in cardiac pericytes from Sham and 4-, 7- and 14-day MI hearts. **(B)** Schematic representation of breeding to generate *Cspg4*$^{CreER/+}$; *Tgfbr1*$^{f/f}$ mice. **(C and D)** Flow cytometric analysis of tdTomato^{+} and tdTomato^{+}/GFP^{+} cells acquired from *Cspg4*$^{CreERT2/+}$*; Tgfbr1*$^{+/+}$ (control) and *Cspg4*$^{CreERT2/+}$*; Tgfbr1*$^{f/f}$ (experimental) mice after 14 days of MI. The Col1a1$^{GFP/+}$ reporter mouse strain drives GFP expression. **(E and F)** *Cspg4*-lineage pericytes and *Col1a1* expressing cells were collected using fluorescence-activated cell sorting (FACS) based on tdTomato fluorescence and were analyzed for expression of *Smad3* between control and experimental mice following 14 days of MI. N=3 samples were analyzed from control and experimental conditions and analyzed using an unpaired two-tailed t-test. **(G)** Immunohistochemical analysis of pericytes (red) and phosphorylated SMAD3 (pSMAD3, green) in control and experimental mice following 14 days of MI**.** Yellow arrows highlight pSMAD3 in tdTomato^{+} pericytes. **(H)** Immunohistochemical analysis of pericytes (red) and ALK1 (green) in control and experimental mice following 7 days of MI**. (I)** Visualization of vascular permeability based on dextran (white) outside of isolectin (green) labeled vasculature. Scale bar: 20μm. A, G, H, and I, Representative of three experiments (sham versus MI and control versus experimental hearts).

Supplemental Figure 8

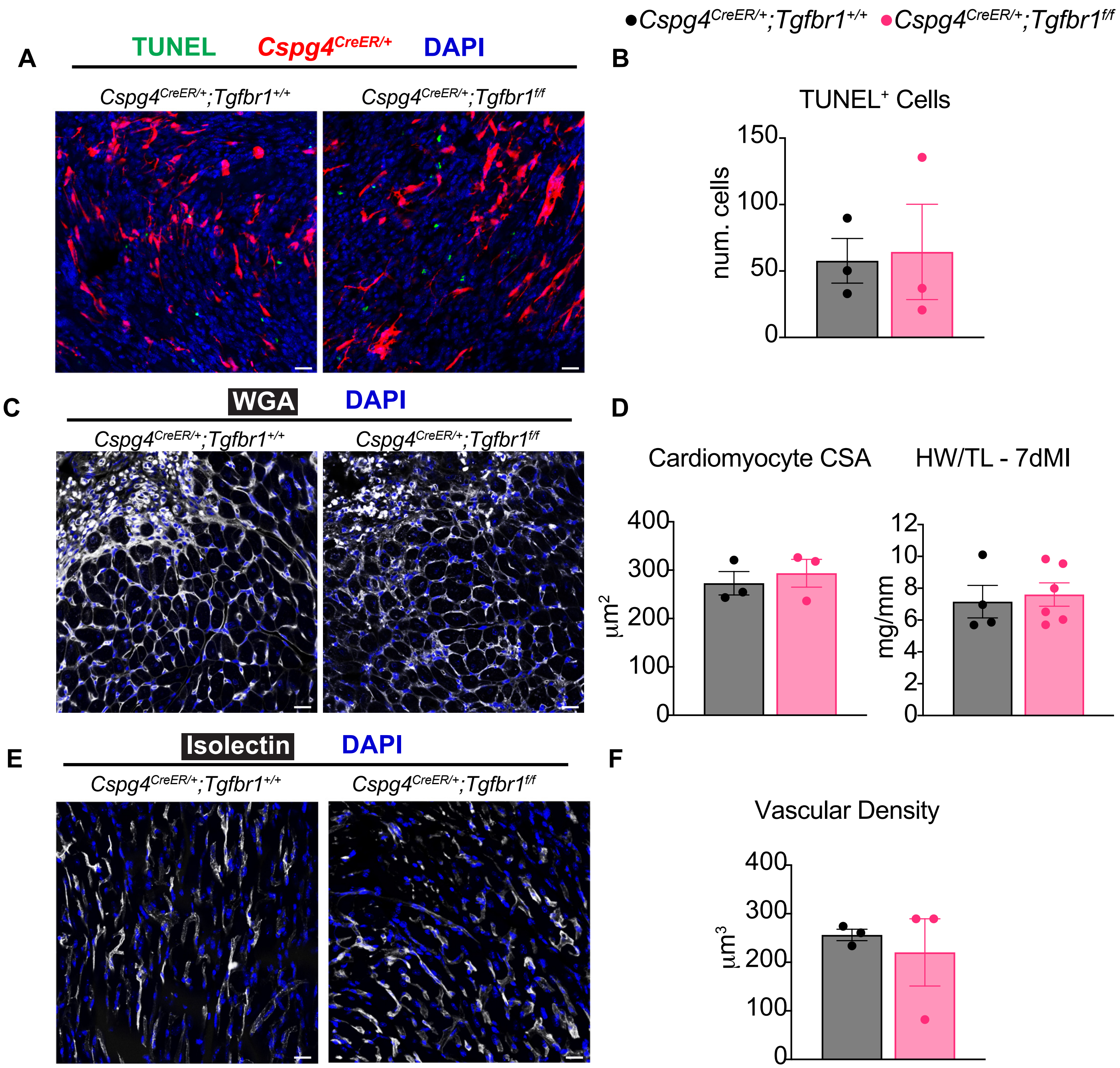

Supplemental Figure 8. Deletion of *Tgfbr1* in *Cspg4*-lineage cells does not alter cell survival, cardiomyocyte size, or vascular density. Analysis of *Cspg4*$^{CreERT2/+}$*; Tgfbr1*$^{+/+}$ (control) and *Cspg4*$^{CreERT2/+}$*; Tgfbr1*$^{f/f}$ (experimental) mice at 7 days post-MI. **(A)** Immunohistochemical analysis of apoptotic cells (TUNEL positive) with **(B)** quantitation represented by the number of cells per field of view. N=3 control and experimental mouse hearts were analyzed using an unpaired two-tailed t-test. **(C)** Immunohistochemical analysis of cardiomyocyte hypertrophy visualized by staining with WGA (white). **(C)** Quantitation of cardiomyocyte cross-sectional area (CSA, μm^2). N=3 control and experimental mouse hearts were analyzed using an unpaired two-tailed t-test. **(D)** Heart weight to tibia length in control and experimental mice following 7 days of MI. N=4 control and N=6 experimental mouse hearts were analyzed using an unpaired two-tailed t-test. **(E)** Immunohistochemical analysis of vascular density visualized by labeling for isolectin-positive vasculature (white). **(F)** Quantitation of vascular density (μm^3). N=3 control and experimental mouse hearts were analyzed using an unpaired two-tailed t-test. Scale bar: 20μm. A, C, and E, Representative of three experiments.

Supplemental Figure 9

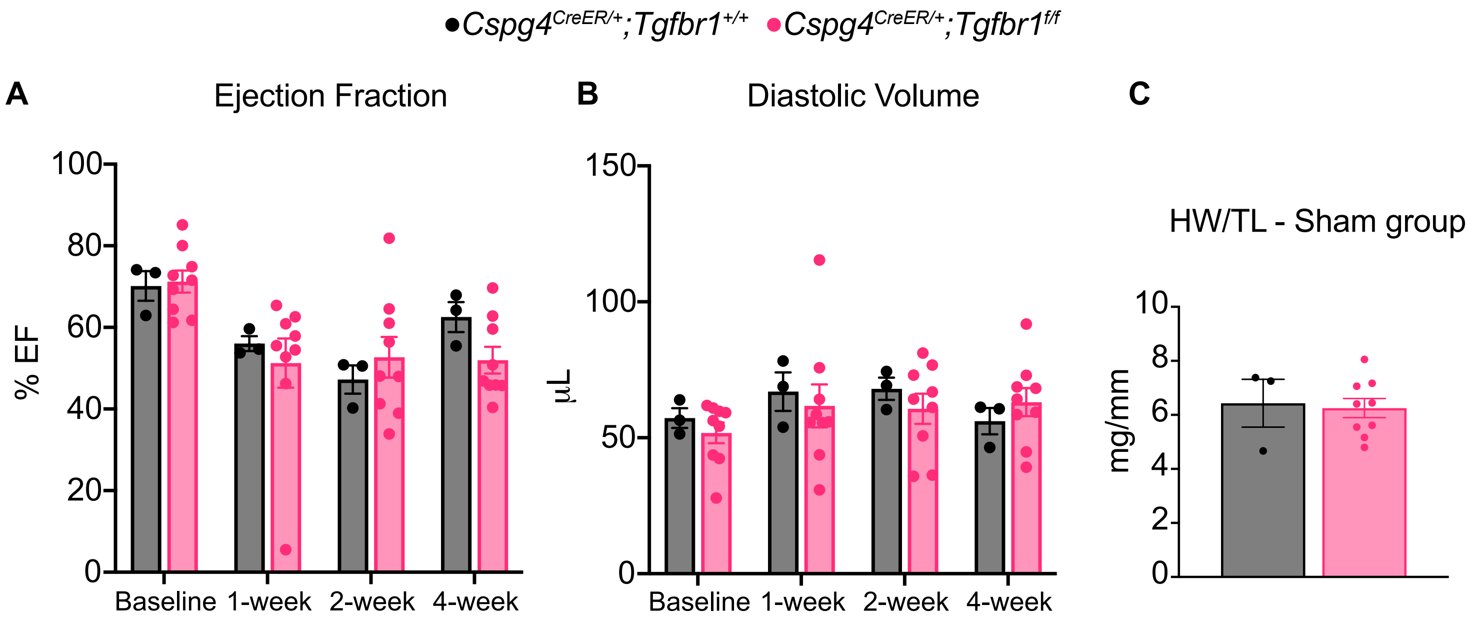

Supplemental Figure 9. Deletion of *Tgfbr1* in *Cspg4*-lineage cells does not alter cardiac function or morphometry following sham surgery. Evaluation of **(A)** cardiac function through measurement of ejection fraction and **(B)** cardiac morphometry by analysis of left ventricular diastolic volume in control *Cspg4*$^{CreER/+}$; *Tgfbr1*$^{+/+}$ and experimental *Cspg4*$^{CreER/+}$; *Tgfbr1*$^{f/f}$ mice. N=3 control and N =9 experimental at baseline, 1-, 2-, and 4-week post-MI. Analyzed using Two-Way ANOVA with Sidak multiple comparisons test. **(C)** Heart weight to tibia length in sham mice following 4 weeks of MI. N=3 control and N =9 experimental at baseline, 1-, 2-, and 4-week post-MI. It was analyzed using an unpaired two-tailed t-test.

Supplemental Figure 10

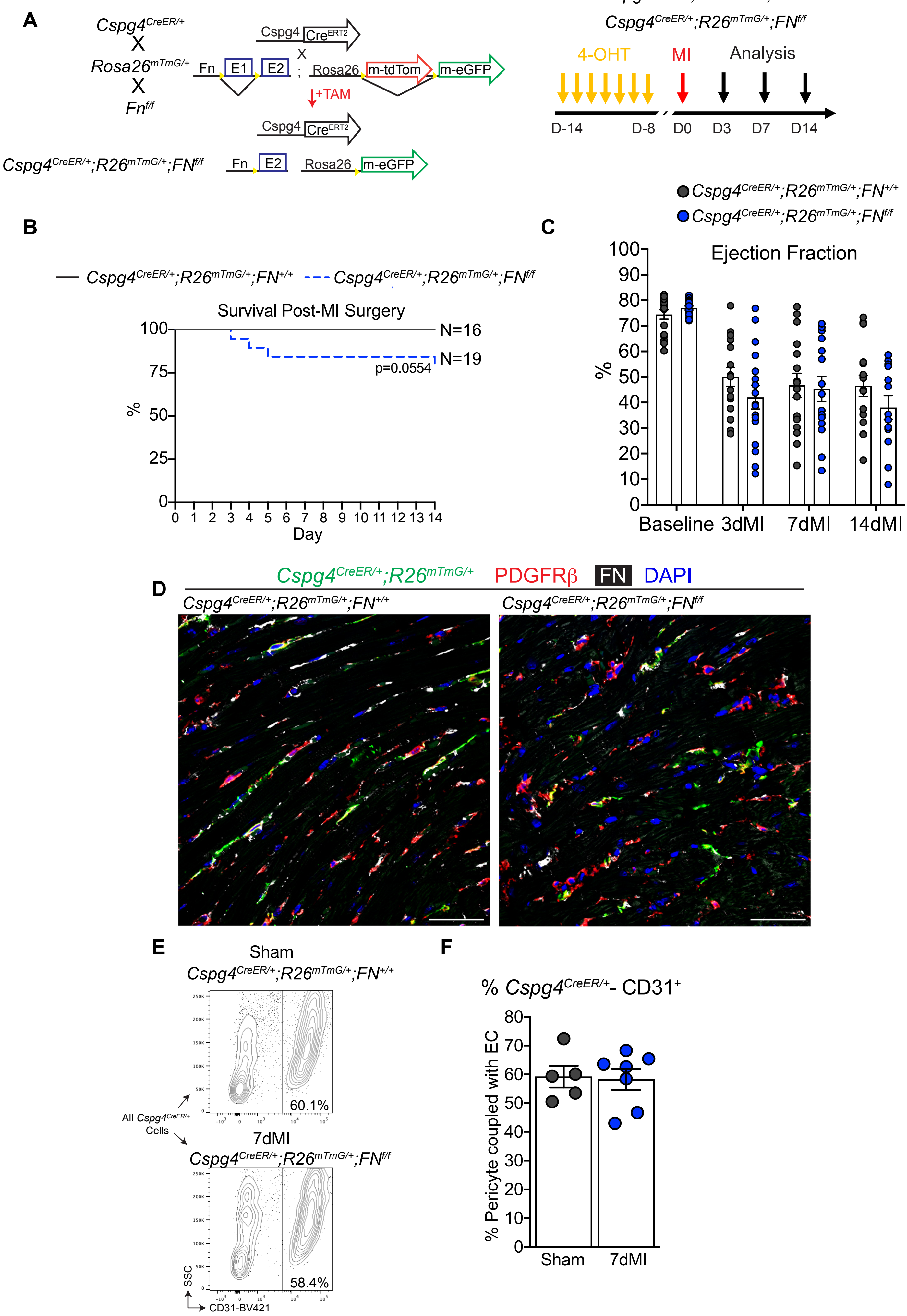

Supplemental Figure 10. Deletion of fibronectin (Fn) in pericytes does not alter cardiac function or dissociation of pericytes from the vasculature. (A) Schematic representation for the generation of *Cspg4*$^{CreERT2/+}$*; R26*$^{mTmG/+}$*; FN*$^{+/+}$ (control) or *FN*$^{fl/fl}$ (experimental) mice before tamoxifen administration and performance of sham or myocardial infarction (MI). **(B)** Kaplan-Meier survival curve of control or experimental mice following sham or MI surgery. N=16 control and N=19 experimental mice were analyzed using Log-rank (Mantel-Cox) test. **(C)** Ejection fraction was measured in sham and MI animals and between control and experimental mice starting at baseline and up to 14 days post-surgery. N=17 control and N=18 experimental at baseline; N=16 control and N=17 experimental at 3 days post-MI; N=16 control and N=15 experimental at 7 days post-MI; N=16 control and N=13 experimental at 14 days post-MI. Analyzed using Two-Way ANOVA with Sidak multiple comparisons test. **(D)** Immunohistochemical analysis of *Cspg4*-lineage pericytes (green) and expression of FN with co-localization of PDGFRβ. Scale bar: 40μm. Representative of three control and experimental hearts. **(E)** Flow cytometric analysis of pericytes associated with CD31^{+} endothelial cells **(F)** Quantitation of *Cspg4*-lineage pericytes with CD31^{+} endothelial cells, which was not significantly altered between control and experimental animals. N=4 control and N=7 experimental and analyzed using an unpaired two-tailed t-test.

Supplemental Figure 11

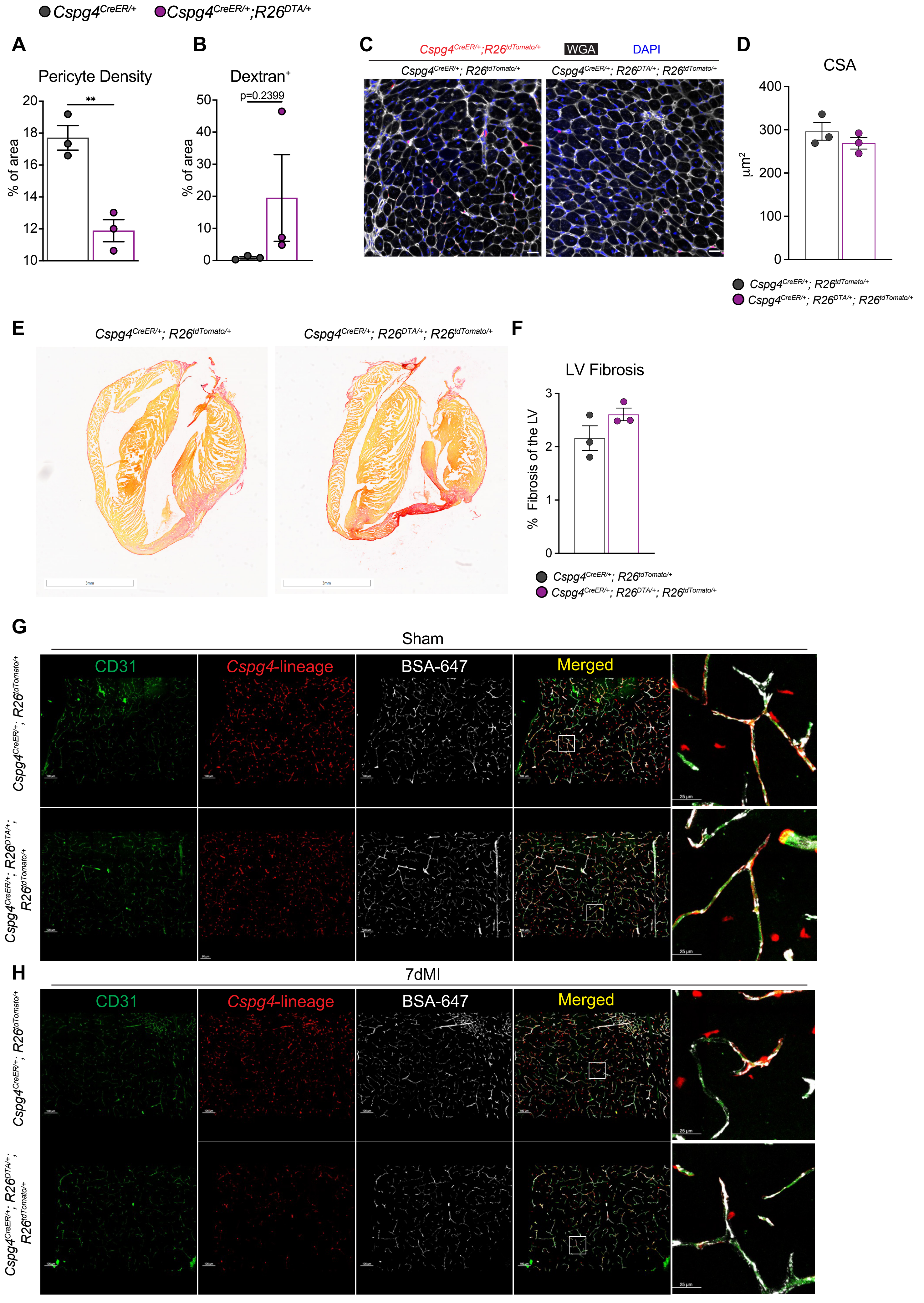

Supplemental Figure 11. Assessment of vascular permeability in the heart and brain following pericyte ablation and myocardial infarction injury. Quantifying **(A)** Pericyte density and **(B)** Dextran extravasation as a percentage of the myocardial area following 7 days of myocardial infarction in control and $Cspg4^{CreER/+}$; $R26^{DTA/+}$ mice. N=3 control and experimental mouse heart were analyzed using an unpaired two-tailed t-test. **(C)** Visualization of wheat germ agglutinin to visualize pericytes (red) and cardiomyocyte size. Scale bar: 40μm. **(D)** Quantitation of cardiomyocyte cross-sectional area measured in $Cspg4^{CreER/+}$; $R26^{tdTomato/+}$ and $Cspg4^{CreER/+}$; $R26^{DTA/+}$; $R26^{tdTomato/+}$ mice following 7 days of ischemia. N=3 control and experimental and analyzed using an unpaired two-tailed t-test. (**E)** Picrosirius Red staining to measure fibrosis in the hearts of $Cspg4^{CreER/+}$; $R26^{tdTomato/+}$ and $Cspg4^{CreER/+}$; $R26^{DTA/+}$; $R26^{tdTomato/+}$ mice following 7 days of ischemia. **(F)** Quantitation of fibrosis in the left ventricular free wall. N=3 control and experimental and analyzed using an unpaired two-tailed t-test. **(G and H)** Coronal section of the anterior hypothalamus in $Cspg4^{CreER/+}$; $R26^{tdTomato/+}$ and $Cspg4^{CreER/+}$; $R26^{DTA/+}$; $R26^{tdTomato/+}$ mice subjected to sham (G) or 7 days of MI (H). Brain vasculature was visualized using the pan-endothelial cell marker CD31, and vascular leakage was visualized with BSA-647. Pericytes of the *Cspg4*-lineage are indicated in red. C and E, Representative of three control and experimental hearts. G, Representative of three control and 2 experimental hearts following sham surgery. H, Representative of three control and experimental hearts following MI. **$p<0.01$.
